# Recovery and Degradation Drive Changes in the Dispersal Capacity of Stream Macroinvertebrate Communities

**DOI:** 10.1111/gcb.70054

**Published:** 2025-01-27

**Authors:** Carlos Cano‐Barbacil, James S. Sinclair, Ellen A. R. Welti, Peter Haase

**Affiliations:** ^1^ Department of River Ecology and Conservation Senckenberg Research Institute and Natural History Museum Frankfurt Gelnhausen Germany; ^2^ Conservation Ecology Center Smithsonian's National Zoo and Conservation Biology Institute Front Royal Virginia USA; ^3^ Faculty of Biology University of Duisburg‐Essen Essen Germany

**Keywords:** dispersal capacity metric, ecological quality ratio, Europe, freshwater macroinvertebrates, restoration, river, time series, traits

## Abstract

Freshwater ecosystems face significant threats, including pollution, habitat loss, invasive species, and climate change. To address these challenges, management strategies and restoration efforts have been broadly implemented. Across Europe, such efforts have resulted in overall improvements in freshwater biodiversity, but recovery has stalled or failed to occur in many localities, which may be partly caused by the limited dispersal capacity of many species. Here, we used a comprehensive dataset comprising 1327 time series of freshwater macroinvertebrate communities ranging from 1968 to 2021 across 23 European countries to investigate whether dispersal capacity changes with the ecological quality of riverine systems. Sites experiencing improvements in ecological quality exhibited a net gain in species and tended to have macroinvertebrate communities containing species with stronger dispersal capacity (e.g., active aquatic and aerial dispersers, species with frequent propensity to drift, and insects with larger wings). In contrast, sites experiencing degradation of ecological quality exhibited a net loss of species and a reduction in the proportion of strong dispersers. However, this response varied extensively among countries and local sites, with some improving sites exhibiting no parallel gains in macroinvertebrates with higher dispersal capacity. Dispersal capacity of the local species pool can affect the success of freshwater ecosystem restoration projects. Management strategies should focus on enhancing landscape connectivity to create accessible “source” areas and refugia for sensitive taxa, especially as climate change reshapes habitat suitability. Additionally, biodiversity initiatives must incorporate adaptive decision‐making approaches that account for the site‐specific responses of macroinvertebrate communities to changes in ecological quality.

## Graphical abstract

Freshwater ecosystems across Europe are recovering unevenly despite restoration efforts, partly due to the limited ability of many species to disperse. Using a dataset of over 1300 time series, we found that improved ecological quality often leads to gains in macroinvertebrate species with strong dispersal capacities, while degrading sites lose these species. Restoration success depends on enhancing habitat connectivity and considering species' dispersal traits, especially in the context of climate change.

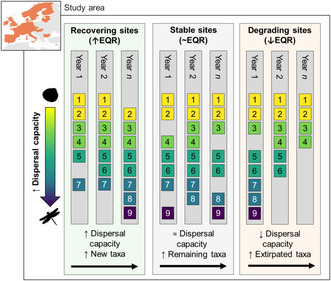

## RESUMEN

Los ecosistemas de agua dulce se enfrentan a importantes amenazas, como la contaminación, la pérdida de hábitats, las especies invasoras y el cambio climático. Para hacer frente a estos retos, se han aplicado ampliamente estrategias de gestión y esfuerzos de restauración. En toda Europa, estos esfuerzos han dado lugar a mejoras generales de la biodiversidad en ecosistemas de agua dulce, pero la recuperación se ha estancado o no se ha producido en muchas localidades, lo que puede deberse en parte a la limitada capacidad de dispersión de algunas especies. En este trabajo, hemos utilizado un amplio conjunto de datos que comprende 1327 series temporales de comunidades de macroinvertebrados de agua dulce desde 1968 hasta 2021 en 23 países europeos para investigar si la capacidad de dispersión de dichas comunidades cambia con la calidad ecológica de los sistemas fluviales. Los lugares con una mejor calidad ecológica experimentaron una ganancia neta de especies y tendieron a tener comunidades de macroinvertebrados que contenían especies con mayor capacidad de dispersión (por ejemplo, dispersores acuáticos y aéreos activos, insectos con alas más grandes). Por el contrario, los lugares más degradados experimentaron una pérdida neta de especies y mostraron una reducción de la proporción de grandes dispersores. Sin embargo, esta respuesta varió ampliamente entre países y lugares, y algunos sitios que mostraron una mejora de la calidad ecológica a lo largo del tiempo no presentaron ganancias paralelas de macroinvertebrados con mayor capacidad de dispersión. La capacidad de dispersión de las especies locales puede afectar al éxito de los proyectos de restauración de ecosistemas de agua dulce. Las estrategias de gestión deben centrarse en mejorar la conectividad del paisaje para crear zonas «fuente» accesibles y refugios para especies sensibles, especialmente a medida que el cambio climático modifica la idoneidad del hábitat. Además, las iniciativas de restauración de la biodiversidad deben incorporar enfoques de toma de decisiones adaptativos que tengan en cuenta las respuestas específicas de las comunidades de macroinvertebrados a los cambios en la calidad ecológica.

## 1 Introduction

Freshwater ecosystems harbor disproportionate levels of biodiversity relative to their spatial extent and provide key goods and services for society, including water for domestic use, industry, agriculture, recreation, energy production, and transportation corridors (Geist 2011; Haase et al. 2023). However, rivers and lakes are among Earth's most altered ecosystems, threatened by overexploitation, water pollution, fragmentation, habitat transformation, biological invasions, and climate change (Grill et al. 2019; Rahel and Olden 2008). National and international protective regulations for freshwater ecosystems, such as the US Clean Water Act (1972) and the EU Water Framework Directive (WFD; 2000), have promoted reductions in metal pollution, organic pollution, and acidification (EEA 2018; Smail et al. 2012). This has led to a partial recovery of freshwater biodiversity during the last 50 years (Haase et al. 2023; Vaughan and Gotelli 2019; White et al. 2017). However, many rivers exhibited no recovery (Sinclair et al. 2024), and most that recovered have reached a plateau, with macroinvertebrate communities remaining stable since around 2010 (Haase et al. 2023). Meanwhile, the impacts and number of stressors affecting freshwater ecosystems continue to increase globally, with rivers' ecological quality remaining poor worldwide (Birk et al. 2020). Thus, it is critical to determine why recovery has not occurred or has halted to promote further improvement in freshwater ecosystems.

Community recovery processes commonly require recolonization, which depends, in part, on species dispersal. Dispersal is an ecological process that involves the displacement of individuals away from their natal habitat to another where they settle and reproduce (Hanski 1999). Species dispersal is an essential process that strongly influences metapopulation and metacommunity dynamics through the movement of individuals and species, respectively (Heino et al. 2015; Massol et al. 2017; Tonkin et al. 2017). Dispersal plays a crucial role in maintaining population connectivity and promoting species persistence in fragmented or disturbed landscapes (Cunillera‐Montcusí et al. 2021). By linking spatially distinct populations and communities, dispersal regulates both local and regional biodiversity, as well as the recovery trajectories of ecosystems undergoing environmental change (Leibold et al. 2004). Thus, species dispersal capacities are crucial factors affecting community responses to disturbances, influencing the rate of recolonization, the composition and functionality of communities, and the success of restoration efforts (Heino et al. 2015; Travis et al. 2013). Available evidence supports that species with a weak dispersal capacity tend to arrive later at new restored habitats compared to species with a strong dispersal capacity (see e.g., Figuerola and Green 2002; Tonkin et al. 2014). In addition, degraded ecosystems often harbor species with limited dispersal capacities, contributing less efficiently to recolonization than well‐preserved habitats (Sundermann, Stoll, and Haase 2011). Consequently, community recovery may be limited in areas that lack strong dispersers or in habitats where dispersal barriers inhibit the recolonization of new species.

Riverine macroinvertebrates, which are commonly used to assess the ecological quality of freshwater ecosystems, are an ideal group to assess responses of community dispersal to changes in ecosystem state as they are diverse, cover a range of dispersal abilities, and occupy freshwater ecosystems spanning a range of highly degraded, to improving, to near pristine conditions. Quantifying dispersal capacity (i.e., a measure of the distance and frequency of an organism's movement among different habitats; Li et al. 2016) is notoriously difficult, but substantial progress has been made in recent years for freshwater species (Cano‐Barbacil, Radinger, and García‐Berthou 2020; Li et al. 2016; Radinger and Wolter 2014; Sarremejane et al. 2020; Schmidt‐Kloiber and Hering 2015; Tachet et al. 2010). Metrics representing the overall dispersal capacity of a macroinvertebrate community have been proposed; such as the “Dispersal Capacity Metric” (*DCM*). This metric reflects combined changes among aquatic active, aquatic passive, aerial active, and aerial passive dispersers and can be a useful tool to evaluate the dispersal capacity of stream macroinvertebrates in sites with changing environmental conditions (Li et al. 2016). In addition to the *DCM*, multiple dispersal‐related biological traits of European freshwater macroinvertebrates have been collated (Sarremejane et al. 2020; Schmidt‐Kloiber and Hering 2015; Tachet et al. 2010), allowing assessment of overall trait composition and the dispersal capacity of a given community (Li et al. 2016; Sarremejane et al. 2020). For example, larger‐bodied taxa and flying insects with bigger wings disperse greater distances than smaller‐bodied and smaller‐winged taxa (see Lancaster, Downes, and Kayll 2024), generally showing greater colonization capacity as females are likely to oviposit farther from their source population (Graham, Storey, and Smith 2017; Jenkins et al. 2007). Similarly, long‐lived taxa and species with higher fecundity and multiple reproductive cycles per year typically participate in more dispersal events during their lifespan and therefore have higher capacity to colonize new habitats (Beckman, Bullock, and Salguero‐Gómez 2018; Stevens et al. 2013). Finally, in macroinvertebrate communities, drifting downstream is an effective way to disperse and colonize new areas (Riel, Der Velde, and De Vaate 2011). This strategy can help drifting taxa to escape and evade unfavorable or changing ecological quality conditions (Wooster and Sih 1995).

Here, we used a large‐scale database of 1327 stream macroinvertebrate time series ranging from 1968 to 2021 across 23 European countries (mean time series length: 18.1 years; mean number of sampling years: 14.8) to investigate the role of overall dispersal capacity (i.e., *DCM*) and specific dispersal traits, in shaping community recovery (i.e., the process through which an ecosystem regains its community structure and function after being disturbed; Kelly and Harwell 1990) and degradation processes (i.e., the process through which an ecosystem loss its community structure and function due to environmental stressors; Vos et al. 2023). We examined changes in *DCM* and dispersal‐related traits among taxa gained versus lost in recovered, degraded, and stable sites. We hypothesized that improvements in ecological quality—measured as the ecological quality ratio (EQR)—would relate to the arrival of taxa with greater dispersal capacity (Li et al. 2016) and would facilitate the establishment of active dispersers, longer lived and larger‐bodied taxa (Barnum, Weller, and Williams 2017; Jenkins et al. 2007). We expected that degradation would be associated with a decline in dispersal capacity and the loss of strong dispersers (Barnum, Weller, and Williams 2017), while stable ecological quality levels would result in no changes in the dispersal capacity of the community. Identifying the linkages between species' dispersal capacity and ecosystem recovery and degradation aid evaluation of the effectiveness of current water quality improvement and habitat restoration strategies, and inform management actions to restore or preserve freshwater ecosystems (Barton et al. 2015; Bowler and Benton 2005).

## 2 Methods

### 2.1 Data Compilation

#### 2.1.1 Community Data

We compiled time series of European riverine macroinvertebrate communities from Haase et al. (2023), complemented by additional time series from Czechia and Lithuania (Sinclair et al. 2024). All time series met the following criteria: (1) included abundance estimates, (2) had ≥ 8 sampling years (not necessarily consecutive) and (3) had consistent sampling methods and effort per site throughout the sampling period. Only one sampling event per year was considered for each time series. Sampling was always conducted during the same season (defined as any three consecutive months). For time series with multiple sampling seasons within or among years, we included only one sampling season, preferably using the season with the longest time series. The majority of time series (81%) exhibited near‐annual sampling (i.e., an average interval between samples of less than 2 years). No substantial differences in this sampling frequency are observed across countries, with the exception of Belgium, where sites were sampled every 2–3 years.

**FIGURE 1:**
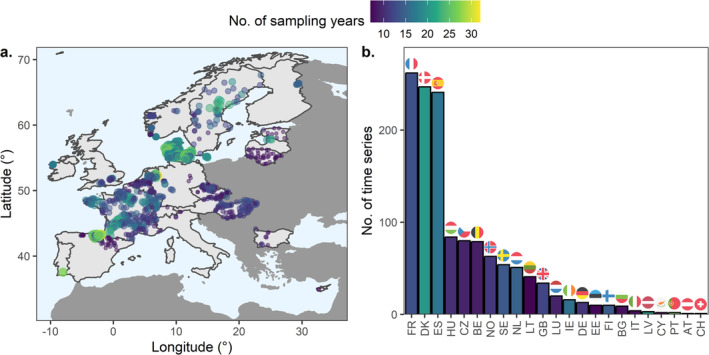
(a) Sampling sites (points) and number of sampling years (color and size of points) across 23 European countries (light gray). (b) Number of time series and mean number of sampling years (color) by country. AT, Austria; BE, Belgium; BG, Bulgaria; CH, Switzerland; CY, Cyprus; CZ, Czechia; DE, Germany; DK, Denmark; EE, Estonia; ES, Spain; FI, Finland; FR, France; GB, United Kingdom; HU, Hungary; IE, Ireland; IT, Italy; LT, Lithuania; LU, Luxembourg; LV, Latvia; NL, Netherlands; NO, Norway; PT, Portugal; SE, Sweden. Map lines delineate study areas and do not necessarily depict accepted national boundaries.

Taxa were primarily identified to family, genus or species levels, although some were classified at intermediate taxonomic resolutions (e.g., Chironominae at subfamily) or higher levels (e.g., Oligochaeta at subclass). The resolutions used in each country are based on their own assessment methods of what is most informative and feasible for capturing community responses to anthropogenic impacts in each region; therefore, the identification level can vary among countries due to differences in national policy (Birk et al. 2012). However, we ensured that the taxonomic resolution was consistent within time series. Taxa names were harmonized using the “freshwaterecology.info” database (Schmidt‐Kloiber and Hering 2015) to correct for inconsistencies within time series (e.g., changes in the nomenclature of a specific taxa over time). In total, 3.9% of the observations were renamed. The final dataset comprises 546,488 observations of 2640 macroinvertebrate taxa in 1327 sites and 23 countries ranging from 1968 to 2021 (Figure 1). The time series span a mean of 18.1 years with an average of 14.8 sampling years (minimum 8 years, maximum 32 years).

#### 2.1.2 Ecological Quality

In Europe, ecological quality is consistently measured in compliance with the EU WFD and quantified as a ratio between the value of the observed biological parameter for a given water body (e.g., composition or species richness) and the expected value under undisturbed conditions (hereafter, reference conditions; see e.g., Mykrä et al. 2012; Pinto, Rodrigues, and Antunes 2021). Reference conditions describe the potentially natural state of a water body at present as it would be without any human interference. These conditions vary depending on the country and the specific river type. In some cases, true references no longer exist (e.g., larger rivers), and are replaced with modelled or collected data from other minimally impacted communities (Sinclair et al. 2024). The EQR provides a common measure that is comparable across countries and different methodologies (see Table S1 for further details on different protocols; Phillips 2014). Higher ratio values indicate communities that have become more similar to reference conditions, while lower ratio values indicate degradation. Here, we quantified the ecological quality of the 1327 studied sites using the EQR (Cano‐Barbacil et al. 2025). In order to harmonize criteria, we transformed the original EQR data by truncating any value > 1 to be equal to 1, which is done by default in certain countries (e.g., Germany; Carstensen et al. 2015; Pereira Coutinho et al. 2012). All time series considered followed a consistent method to calculate EQR throughout the sampling period. In some cases, we restricted the original dataset to ensure this consistency. For example, in Germany, we only included data collected after 2000, when the WFD was implemented, as their sampling methods were updated to comply with the new official standards.

#### 2.1.3 Dispersal Traits

We obtained trait data on European macroinvertebrates from a recently published database that encompasses the most significant traits associated with dispersal abilities (Sarremejane et al. 2020), complemented with data from “freshwaterecology.info” (Schmidt‐Kloiber and Hering 2015). This dataset covered nine dispersal‐related traits divided into 40 categories (see the complete list in Table S2). Trait information was expressed as proportions (i.e., the sum of all categories within each trait sums to one). We imputed missing data using the mean of the available trait values within the same genus, when available, or alternatively within the same family. Consequently, the average data availability of the dataset was enhanced from 44.4% (varying from 34.4% to 48.9% within individual traits) to 92.9% (78.1%–97.1%). We calculated the dispersal capacity metric for each taxon (*DCM*_*s*_) as proposed by Li et al. (2016), which provides an overall estimate of the ability to disperse of freshwater macroinvertebrates, and is calculated as follows:

$${\mathrm{DCM}}_{s}=\frac{\left({\mathrm{aqa}}_{i}+{\mathrm{aqp}}_{i}+2\times {\mathrm{aea}}_{i}+2\times {\mathrm{aep}}_{i}\right)-\min_{c}}{\max_{c}-\min_{c}}$$

where *aqa*_*i*_ is the aquatic active dispersal strategy of taxa i, *aqp*_*i*_ is the aquatic passive dispersal strategy, *aea*_*i*_ is the aerial active dispersal strategy, and *aep*_*i*_ is the aerial passive dispersal strategy; min_*c*_ and max_*c*_ are the taxa with the lowest and the highest sum of dispersal capacity values within the entire community *c*, respectively.

### 2.2 Statistical Analysis

We calculated the community‐weighted means (CWMs; i.e. average trait value of a community, weighted by the relative abundance of each taxa present; Madrigal‐González et al. 2020) of the *DCM*_*s*_ and of each dispersal‐related trait category (Cano‐Barbacil et al. 2025). We first used generalized additive mixed models (GAMM) of the beta family to evaluate the relationship between CWM‐*DCM* and EQR values. The model was implemented using the *gamm* function of the *mgcv* package (Wood 2023) and included EQR as a smoothed fixed effect which varied by country, while considering year as a smoothed random intercept effect. The basis dimension (*k*) was set to 10, which we confirmed by using the *gam.check* function of the *mgcv* package. We accounted for temporal autocorrelation (Figure S1) by including first‐order autocorrelation between successive years sampled from the same site.

We used linear models and calculated the slopes of the EQR and CWM‐*DCM* to evaluate changes through time for each time series. Response variables were logit‐transformed in both models, as they range between 0 and 1 (Cano‐Barbacil et al. 2020; Warton and Hui 2011). We used linear trends to categorize sites into three distinct groups based on their changes on EQR and CWM‐*DCM* over time: (1) recovering sites with a positive trend (positive slope and *p* < 0.05), (2) stable sites with no trend (*p* > 0.05), and (3) degrading sites with a negative trend (negative slope and *p* < 0.05). We constructed a contingency table to summarize the relationship between pairs of groups, calculated the corrected contingency coefficient *C*_corr_ using the function *ContCoef* of the R‐package *DescTools*, and performed a *χ* ^2^ test to measure the association between changes in CWM‐*DCM* and changes in the EQR (Cano‐Barbacil, Olden, and García‐Berthou 2024; Signorell 2023).

To evaluate if an increase in dispersal capacity of the community is related to an increase in ecological quality, or if a decrease is related to environmental degradation, the resulting slope estimates were used to determine whether the CWM‐*DCM* slopes were correlated to the EQR slopes. For this purpose, we used a linear mixed model conducted using the *lme4* package (Bates et al. 2015). In this model, both a random intercept term and a random slope term for country were included. Country was used as the grouping factor to account for differences in sampling protocols and the implementation of varying water management policies across regions (Sinclair et al. 2024). We tested the need for inclusion of the random effect term in the model using the *ranova* function of the *lmerTest* R‐package (Kuznetsova, Brockhoff, and Christensen 2017) to see if the relationship between dispersal capacity and ecological quality differed across countries. We calculated marginal and conditional *R* ^2^ values with the *r.squaredGLMM* function of the *MuMIn* R‐package (Barton 2023). Marginal *R* ^2^ describes the variability explained by the fixed effects, while the conditional *R* ^2^ describes the variability jointly explained by the fixed and the random effects. We repeated this analysis for the rest of the dispersal traits and corrected the *P* values for multiple‐comparisons using the false discovery rate (FDR) with the R function *p.adjust* (Benjamini and Hochberg 1995; Saberi and Seyed‐allaei 2016).

Finally, we used the first and final 3 years of each time series to identify which taxa were “new” (not present initially and then present later), “remaining” (no change), and “extirpated” (present and then not present) within each community (see e.g., Louhi et al. 2011), and to determine whether new taxa have higher or lower dispersal capacity than extirpated taxa. To compare their dispersal capacity, we calculated the mean *DCM* of these three taxa groups among sites with increasing, stable, and decreasing EQR. For this purpose, we used a linear mixed model including site as random intercept; again we tested the need for inclusion of the random effect term in the model and calculated the marginal and conditional *R* ^2^. Because the first and last three sampling years in each time series were not always consecutive, we re‐ran this analysis including only sites where the first and last 3 years were consecutive. The resulting dataset comprises data from 506 sites and 20 countries. This approach helps account for species that may have been temporarily absent or undetected in non‐consecutive years, but still present in the ecosystem. By focusing on consecutive years, we aimed to reduce the risk of misclassifying such species as “new” or “extirpated” due to irregular sampling intervals. The results of this analysis, which were consistent with those from the full dataset, are summarized in Appendix S1.

All aforementioned analyses were repeated for each dispersal‐related trait. This included calculating the slopes for each trait over time and evaluating their relationship with changes in ecological quality for each site and comparing the traits of new, remaining, and extirpated taxa in recovering, stable, and degrading sites. All statistical analyses were carried out with the software R version 4.2.2 (R Core Team 2023).

## 3 Results

### 3.1 Ecological Quality Drives Changes in Dispersal Capacity and Traits

**FIGURE 2:**
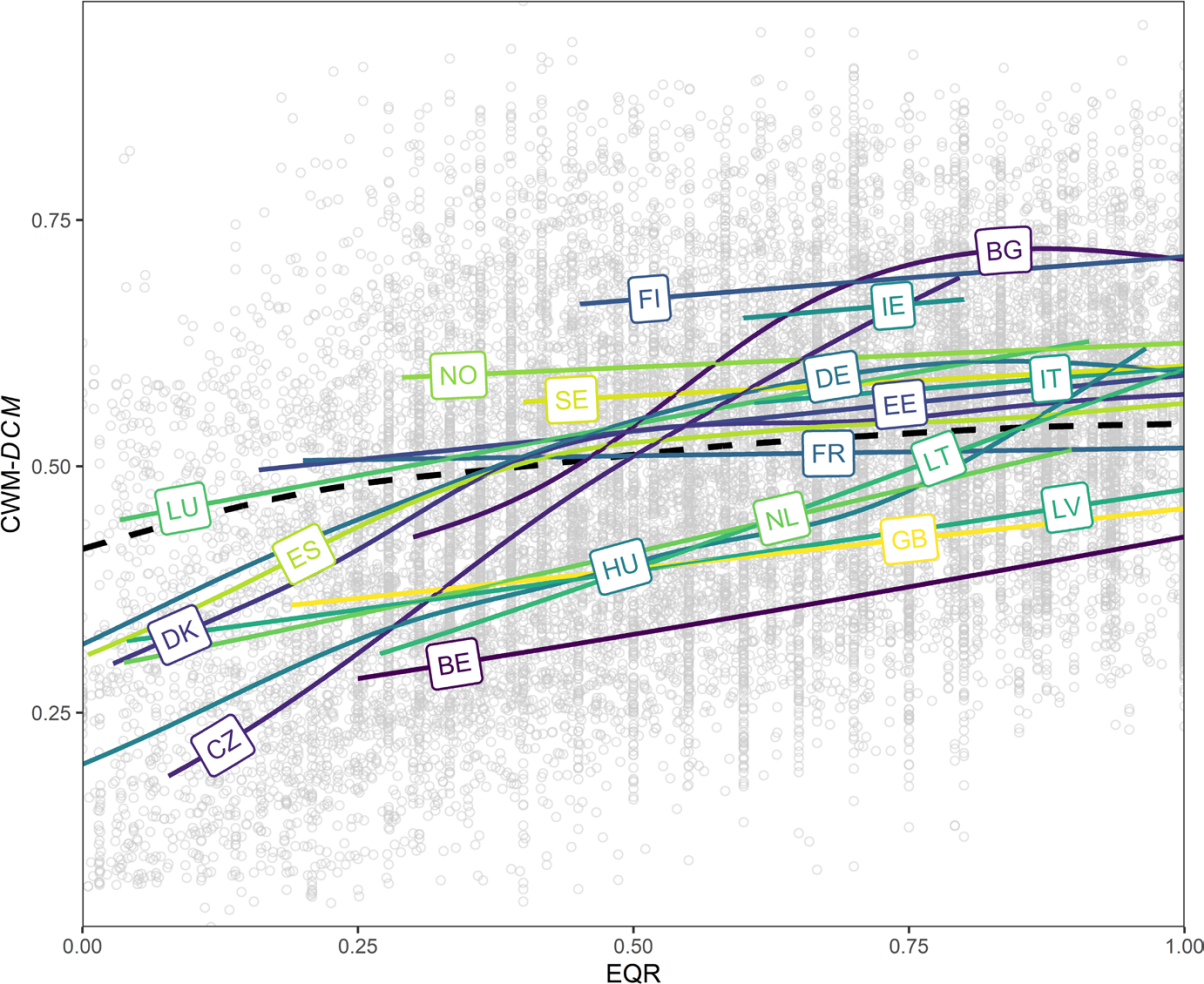
Relationship between the dispersal capacity metric of the community (CWM‐*DCM*) and the ecological quality ratio (EQR). Higher EQR values indicate communities that have become more similar to reference conditions, while lower values indicate degradation. Data points are represented by gray circles and fitted models by solid lines. The colored lines correspond to a model where the smooth term was replicated for each country. The black dashed line corresponds to a model in which a single slope but different intercepts were fitted to all countries and is represented here with the mean intercept. Note that trends for countries with less than three sites with long enough time series were not represented, although they were included when calculating the overall trend. BE, Belgium; BG, Bulgaria; CZ, Czechia; DE, Germany; DK, Denmark; EE, Estonia; ES, Spain; FI, Finland; FR, France; GB, United Kingdom; HU, Hungary; IE, Ireland; IT, Italy; LT, Lithuania; LU, Luxembourg; LV, Latvia; NL, Netherlands; NO, Norway; SE, Sweden.

Overall, we found that community dispersal capacity (CWM‐*DCM*) increases with ecological quality (EQR) (*R* ^2^ = 33.8%; Figure 2). However, the relationship between EQR and CWM‐*DCM* was non‐linear, with a stronger positive overall slope at lower EQR values (e.g., slope_0.1_ = 0.292; slope_0.3_ = 0.121) that flattened as EQR increased (e.g., slope_0.6_ = 0.089; slope_0.8_ = 0.058; Figure 2). Moreover, the relationship between EQR and CWM‐*DCM* varied significantly among European countries. For instance, the studied macroinvertebrate communities in Belgium or Czechia, among others, showed a strong positive linear relationship between EQR and CWM‐*DCM*, while this relationship was non‐linear in the studied communities from other countries such as Spain, Denmark, and Germany. Similarly, communities in other countries such as Norway had a weak positive linear relationship between EQR and CWM‐*DCM*.

**FIGURE 3:**
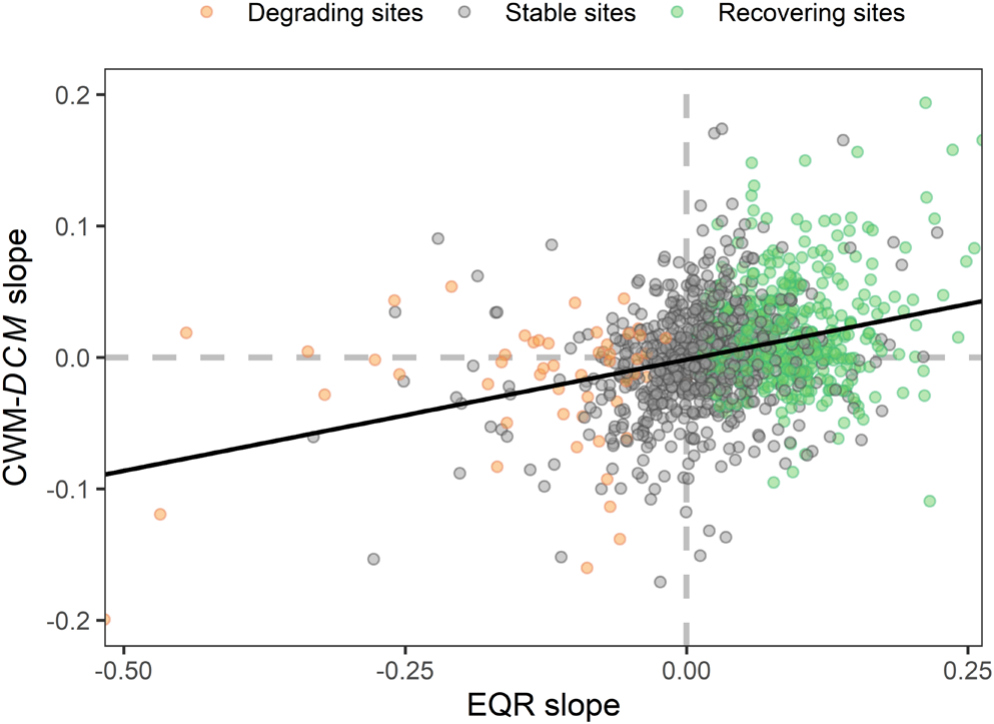
Relationship between changes over time in the dispersal capacity metric of the community (CWM‐*DCM* slope) and changes in the ecological quality ratio (EQR slope). Green dots represent recovering sites, gray dots represent stable sites, and orange dots represent degrading sites. The black line corresponds to a linear model in which a single slope was fitted to all countries. Recovering sites: Increasing ecological quality (*n* = 433), stable sites: No significant changes in ecological quality (*n* = 842), and degrading sites: Decreasing ecological quality (*n* = 52).

Linear models revealed that 32.6% of the studied sites showed significant positive temporal trends in EQR, while only 3.9% showed negative trends (Figure S2a). Similarly, 13.2% of the time series showed increasing dispersal capacity of the macroinvertebrate communities (i.e., CWM‐*DCM*), against the 8.4% that showed negative trends (Figure S2b). We found that changes in CWM‐*DCM* were significantly associated with changes in EQR (*C*_corr_ = 0.347, *χ* ^2^ = 115.9, df = 4, *p* < 0.001; Table 1). This result was supported by the linear mixed model, which revealed a positive relationship between changes in the dispersal capacity (i.e., CWM‐*DCM* slope) and changes in EQR (i.e., EQR slope) (coef. = 0.172, SE = 0.049, df = 15.2, *p* = 0.003; Figure 3 and Table S3). This implies that higher rates of recovery and degradation resulted in more substantial increments and reductions in the CWM‐*DCM*, respectively, while sites with stable EQR values tend to show minimal changes in the CWM‐*DCM*. Changes in EQR explained 8.7% of the variation (marginal *R* ^2^), while the variation explained when including random effects (i.e., country) increased up to 26.7% (conditional *R* ^2^), highlighting high heterogeneity of slopes across European countries (*P*_rand_ < 0.001; see Figure S3). For instance, relationships between slopes were strongly positive for the studied communities in countries such as Czechia, Lithuania, Hungary, or Spain (coef._CZ_ = 0.623, coef._LT_ = 0.368, coef._HU_ = 0.323, coef._ES_ = 0.183) and weak for Norway or Denmark (coef._NO_ = 0.032, coef._DK_ = 0.015). Bulgaria was the only country where the studied communities had a negative relationship between the slopes of CWM‐*DCM* and EQR (coef._BG_ = −0.046).

**TABLE 1:** Contingency table showing the interrelation between changes in the ecological quality ratio (EQR) and dispersal capacity metric of the community (CWM‐*DCM*). The corrected contingency coefficient *C*_corr_ and the results of the *χ* ^2^ test are shown.

		CWM‐*DCM*			*C*_corr_	*χ* ^2^	df	*p*
		Sites with a positive trend	Sites with no change	Sites with a negative trend				
EQR	Recovering sites	118	289	26				
	Stable sites	57	708	77	0.347	115.9	4	< 0.001
	Degrading sites	0	44	8				

**FIGURE 4:**
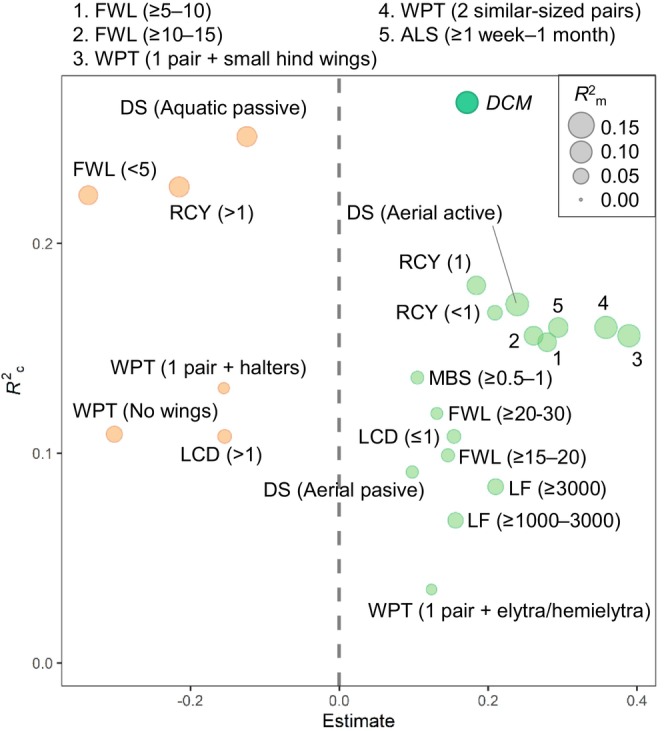
Estimates of the dispersal capacity metric (*DCM*) and dispersal‐related traits of stream macroinvertebrate communities to changes in the ecological quality ratio (EQR). Traits positively correlated to increments in EQR are indicated by green bubbles and negative responses by orange bubbles; non‐significant trait responses are not shown. Slope estimates of CWM slope vs. EQR slope (horizontal axis), conditional *R* ^2^ (vertical axis), and marginal *R* ^2^ (bubble size) are shown for each linear mixed model. ALS, adult lifespan; DS, dispersal strategy; FWL, female wing length (mm); LCD, life‐cycle duration (years); LF, lifelong fecundity (number of eggs per female); MBS, maximum body size (cm); RCY, potential number of reproductive cycles per year; WPT, wing pair type.

Similarly, the proportion of a given community that comprised insects with small (< 5 mm) wings, no wing pairs, or one pair with halters (e.g., Diptera), taxa with more than one potential number of reproductive cycles per year, aquatic passive dispersers, and species with a life‐cycle duration greater than 1 year, all declined as EQRs increased (Figure 4 and Table S3). In contrast, the abundance of middle‐sized taxa (0.5–1 cm), insects with middle‐sized wings (5–30 mm), insects with two similar‐sized pairs of wings (e.g., Odonata), with one pair and elytra (e.g., Coleoptera), or with one pair and small hind wings (e.g., Ephemeroptera) increased as EQRs improved. We found a positive correlation between increases in EQR and increments of short‐lived taxa (< 1 year), taxa with an adult lifespan from 1 week to 1 month, taxa with high fecundity (≥ 1000 eggs per female), taxa with one or less potential number of reproductive cycles per year, and both active and passive aerial dispersers. Changes in EQR explained 3.2% of the variation (marginal *R* ^2^) in changes in dispersal traits on average, while including the random effect (i.e., country) increased the variation explained up to 12.0% (conditional *R* ^2^; see Table S3). However, the explained variation varied substantially among different traits, with insects with two similar‐sized pairs of wings, insects with one pair and small hind wings, and aerial active dispersers all having a stronger positive association with increases in EQR over time (*R* ^2^_m_ = 10%–10.6%).

### 3.2 Dispersal Capacity of New, Remaining, and Extirpated Taxa

**FIGURE 5:**
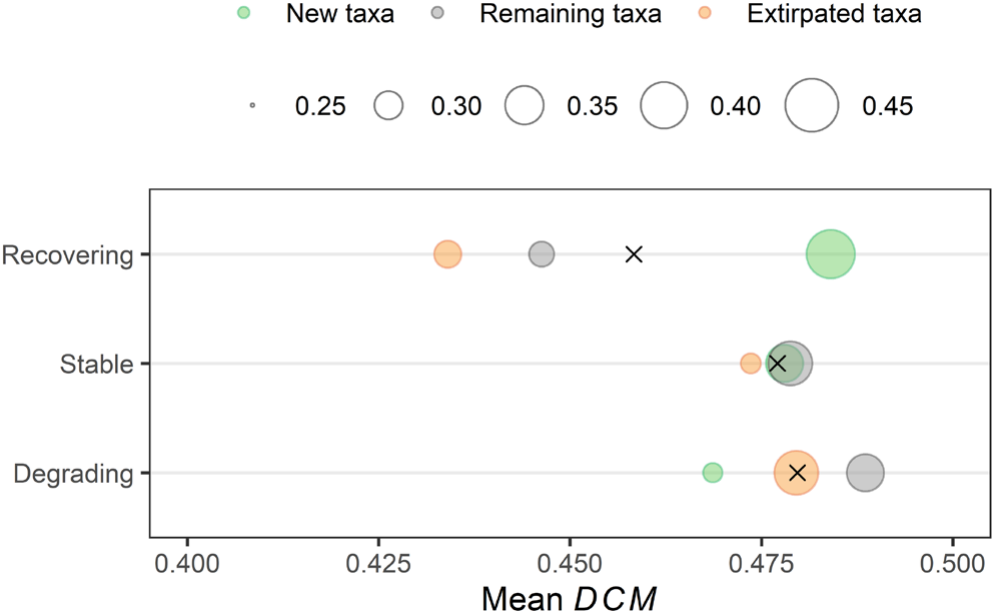
Mean dispersal capacity metric (*DCM*) of new taxa: Not present initially and then present later, remaining taxa: No change and extirpated taxa: Present and then not present in recovering sites: Increasing ecological quality (*n* = 433), stable sites: No significant changes in ecological quality (*n* = 842) and degrading sites: Decreasing ecological quality (*n* = 52). Bubble size indicates the average proportion of new, remaining and extirpated taxa in each site category. Black crosses indicate the weighted mean *DCM* value for each site category. See Figure S4 for further details.

Recovering sites (i.e., increasing EQR) experienced a net gain in taxa due to the arrival and establishment of new taxa, while degrading sites showed a net loss of taxa. Specifically, 41.5% of the taxa recorded in the last three sampling years at recovering sites were new compared to the first three sampling years, while 28.8% remained the same and 29.6% disappeared, on average (Figure 5). Similarly, in stable sites, 34.4% of the taxa were new, 38.4% remained the same, and 27.2% disappeared. In degrading sites, only 27.1% of the taxa were new, while 34.4% remained unchanged and 38.5% disappeared. Overall, we found that taxa with stronger dispersal capacity tended to replace taxa with weaker dispersal capacity in sites where EQR increased over time, while the opposite pattern occurred where EQR decreased. More specifically, new taxa in recovering sites had a higher mean *DCM* than new taxa in degrading sites. Similarly, extirpated taxa in recovering sites had a lower mean *DCM* compared to higher *DCM*s of extirpated taxa in degrading sites (interaction *p* < 0.001; see Figure 5 and Table S4). In sites with no clear EQR trends, new, remaining, and extirpated taxa showed similar mean *DCM*. The linear mixed model revealed that fixed effects explained 3.5% of the variation (marginal *R* ^2^), while the variation explained including random effects (i.e., site) was 27.2% (conditional *R* ^2^), highlighting heterogeneous responses across sites (*P*_rand_ < 0.001).

Similarly, our results demonstrated pervasive, although often small, differences among new, remaining, and extirpated taxa's traits in sites with different EQR temporal trends (Table S4). New species in recovering sites showed traits reflecting higher dispersal capacity (e.g., active aquatic and aerial dispersers, species with frequent propensity to drift, and insects with two similar‐sized pairs of wings or with larger wings) than new taxa in degrading sites (see Tables S4 and S5). In contrast, extirpated taxa in recovering sites generally exhibited traits reflecting lower dispersal capacity (e.g., aquatic passive dispersers, insects with small wings, insects without wings or with one pair of wings and a pair of halters, and taxa with rare propensity to drift) compared to extirpated taxa in degrading sites. Fixed effects explained 3.3% of the variation (marginal *R* ^2^) in dispersal‐related traits on average, while the variation explained with the model also including random effects (i.e., site) increased up to 20.3% (conditional *R* ^2^; see Table S4).

## 4 Discussion

Using a comprehensive pan‐European dataset covering large climatic gradients and 1327 stream macroinvertebrate time series spanning 54 years, we demonstrate that increases in ecological quality—measured as the EQR—typically result in macroinvertebrate communities with higher overall dispersal capacities, favoring the dominance of species with traits that promote strong dispersal such as aerial active dispersers and flying insects (e.g., taxa with one pair of wings and one pair of elytra/hemielytra such as Coleoptera) (Coccia et al. 2021; Lu et al. 2021; Sargac et al. 2021). Higher rates of recovery led to more substantial increments in the dispersal capacity of the community, supporting our hypothesis. Thus, sites with stable EQR values tended to show minimal changes in the dispersal capacity of the community, in line with Li et al. (2016) who reported no significant trends in the dispersal capacity in unrestored sites in Germany. In contrast, degrading sites contained macroinvertebrate communities with lower overall dispersal capacity and species with traits associated with limited dispersal abilities (e.g., species with no wings and aquatic passive dispersers).

### 4.1 Dispersal Capacity of New, Remaining, and Extirpated Taxa

Recovering sites, indicated by increasing ecological quality, demonstrated net gains in species driven by the arrival and establishment of new taxa with higher dispersal capacity and the extirpation of a few species with lower dispersal capacity. The observed arrival of strong dispersers suggests that species with higher dispersal capacity are able to colonize restored sites after short periods of time (Li et al. 2016; Williams 1981). Insect taxa with high dispersal capacity and an aerial active strategy (e.g., Odonata, Trichoptera, or Ephemeroptera) colonize new habitats within months up to a few years (Gergs et al. 2016; Solimini et al. 2003). In particular, adult dragonflies and damselflies have larger and highly aerodynamic wings, efficient flight muscles, and high flight muscle to body mass ratios allowing long distance movements (Marden 1987; Rundle, Bilton, and Foggo 2007). In turn, weakly dispersing non‐insect taxa (e.g., Oligochaeta, Turbellaria, Hirudinea, Gastropoda, and Crustacea) usually require several years to colonize if not moved by vectors including humans and other animals (Gergs et al. 2016; Milner et al. 2008; Minshall, Andrews, and Manuel‐Faler 1983; Winking et al. 2014).

Contrastingly, degrading sites showed a net loss of species, with a substantial number of species being lost and only a few being gained. In these sites, we observed the loss of species with intermediate *DCM* values, which may suggest that these taxa are generally more sensitive to anthropogenic impacts and organic pollution than weak dispersers (Teller, Miller, and Shea 2015; Van Den Berg et al. 2019; see Figure S5). For example, some macroinvertebrate groups with intermediate to high dispersal capacity such as Ephemeroptera, Plecoptera, Trichoptera, and Coleoptera, particularly in the family Elmidae, tend to be less tolerant (Berger et al. 2018), containing taxa that are sensitive to pesticides and other contaminants due to their physiological adaptations (Biggs et al. 2007; Compin and Céréghino 2003). Previous studies have shown that these taxa exhibit marked responses to stressors such as temperature and ion fluctuations, which can impact their overall resilience to environmental changes (see, e.g., Cañedo‐Argüelles et al. 2012; Timoner et al. 2020). For some macroinvertebrates with strong dispersal capacity (e.g., Odonata), an alternative explanation to these extirpations is that individuals are escaping adverse local environmental conditions and dispersing in search of more suitable sites to establish new populations (Khelifa et al. 2021; Propst et al. 2020). However, it is challenging to determine whether active dispersers are leaving degraded habitats or simply experiencing increased mortality. In degrading sites, we also observed a reduced colonization of taxa with low dispersal capacity and some opportunistic features, such as high lifelong fecundity and more than one reproductive cycle per year. Agricultural activities and elevated concentration of nutrients in streams can also cause changes in macroinvertebrate communities, favoring the establishment of weak dispersers and opportunistic species (e.g., Oligochaeta) better adapted to unpredictable environments (Zhang et al. 2021). We found that remaining species in degrading sites have higher dispersal capacity than both new and extirpated taxa, suggesting high dispersal capacity may be beneficial for stable occurrence under occasional unfavorable ecological quality conditions (Gray and Arnott 2011; Wooster and Sih 1995).

Finally, in stable sites, the majority of taxa tend to remain, with only a few gained or lost, suggesting that stable ecological quality generally leads to small changes in community metrics (e.g., richness, diversity, and abundance) (Sinclair et al. 2024). In these sites, the three groups of species studied (i.e., new, remaining, and extirpated species) appear to have similar intermediate dispersal capacity and dispersal‐related traits (Li et al. 2016).

### 4.2 Spatial Variability

While improved ecological quality generally resulted in communities with strong dispersal capacity, this response varied extensively among the studied communities. The heterogeneous response observed among countries could be due to differences in the starting points (i.e., initial EQR values). As shown by our results, the relationship between EQR and CWM‐*DCM* is not linear, with a stronger positive relationship at lower EQR values. Thus, studied communities in countries such as Spain, Lithuania, Hungary, the Netherlands, and Czechia, which generally had lower EQR values than northern countries such as Finland and Sweden (see also Sinclair et al. 2024), showed more pronounced increases and decreases in *DCM* related to recovery and degradation processes, respectively.

At the site scale, we found that many communities with improving ecological quality have not gained macroinvertebrates with higher dispersal capacity. This means that, regardless of improved environmental conditions, macroinvertebrate communities showed weak or even no biotic response to improving habitat in some areas (Dolph et al. 2015; Lorenz et al. 2018; Louhi et al. 2011; Stanford et al. 2020; Wright‐Stow and Wilcock 2017). These heterogeneous recolonization responses suggest that many strong dispersers may be regionally extinct with no nearby source population to support their recolonization, even where habitat conditions have improved (Brederveld et al. 2011; Stoll et al. 2013; Sundermann, Stoll, and Haase 2011; Tonkin et al. 2014; Trekels, Van de Meutter, and Stoks 2011). This could be the case for some species of stoneflies (Plecoptera) in Czechia (Bojková and Soldán 2013) and some water bugs (Hemiptera) in Belgium (Lock et al. 2013) that went regionally extinct in recent decades. Heterogeneous responses at the site scale could also be attributed to other interacting factors such as interspecific competition, dispersal barriers, or parasite loads (Grabner, Schertzinger, and Sures 2014; Parkyn and Smith 2011). For instance, the early arrival and dominance of tolerant species in restored sites can inhibit the colonization of later arriving taxa through competitive exclusion (e.g., Fukami et al. 2005). Competition for space and resources is known to induce a reduction in Ephemeroptera, Plecoptera, and Trichoptera colonization success in restored waterways (Hornblow 2021). Similarly, dams and weirs hinder the upstream dispersal of macroinvertebrate aquatic stages (Sondermann et al. 2017), while extreme topographic relief (e.g., mountain ranges) can act as a dispersal barrier, even for aerial dispersers (Cano‐Barbacil, Radinger, and García‐Berthou 2022; Tonkin et al. 2017). Additionally, the presence of certain parasites can alter the drifting frequency of macroinvertebrates, which can influence both population persistence and dispersal capacity (Humphries and Ruxton 2003; Vance and Peckarsky 1997).

### 4.3 Conservation Implications

Our results suggest that overall community dispersal capacity and dispersal‐related traits emerge as important features influencing population survival and successful colonization of restored or altered freshwater ecosystems. Accordingly, macroinvertebrate communities with greater dispersal capacity and more dispersal‐related traits are expected to be more resilient to climate change and extreme weather events such as floods or droughts, and to catastrophic pollution episodes (Li et al. 2016; Sarremejane et al. 2021; Travis et al. 2013). In order to limit the effects of human‐induced habitat degradation and climate change, it is necessary to implement effective river restoration projects aimed at recovering macroinvertebrate communities, especially taxa with strong dispersal capacities.

Our findings suggest that river management plans would benefit from identifying the causes of the heterogeneous recolonization responses observed to improve key ecosystem functions and ecological processes at sites where macroinvertebrate community recovery has failed. In this context, adaptive decision‐making approaches and long‐term biodiversity monitoring activities are fundamental to identify effective restoration actions and to reassess management alternatives and project goals (Haase et al. 2018; Kail et al. 2015; Maasri et al. 2022; Williams and Brown 2014). For instance, in cases where dispersal barriers or a reduced regional species pool inhibit the arrival of strong dispersers, management efforts should focus on improving landscape connectivity and creating accessible networks of in‐stream refugia (Brederveld et al. 2011; Bush and Hoskins 2017; Markovic et al. 2017), such as through hydromorphological restoration efforts to promote natural flow regimes (e.g., dam removal, reopening of culverted watercourses, remeandering, and reconnection of side arms; Jansson, Nilsson, and Malmqvist 2007; Stromberg et al. 2007). If no response is observed following restoration actions, managers can consider including additional measures that favor the permeability of dispersal barriers to enhance species reintroductions (Clinton et al. 2022; Jourdan et al. 2019; Olden et al. 2011), such as translocations of founder individuals (Baur 2014).

### 4.4 Research Limitations and Future Directions

Although our results are supported by a robust dataset and align with previous studies (see, e.g., Li et al. 2016), we note several limitations. First, our analyses are spatially restricted to only river sites for which we obtained data that met our requirements. Countries such as France, Denmark, Lithuania, and Hungary are well‐represented, with sampling points spread across broad geographic areas. In contrast, countries like Germany, Italy, Portugal, Spain, and Finland have sampling points concentrated in specific basins and regions. As a result, findings in these countries may only reflect conditions in the sampled areas, rather than representing national contexts. Secondly, our analyses were temporally restricted, as reliable monitoring data was primarily available from the early 1990s onward. Thus, our results mostly reflect community changes over the past 30 years.

Third, differences in taxonomic resolution across sites, including the inability to classify certain taxa at lower taxonomic levels (e.g., genus or species), may obscure community changes in certain sites. Finally, fine‐scale processes and local factors influencing community dynamics (e.g., habitat type, presence of anthropogenic stressors, or dispersal barriers) may not have been fully captured in our analysis. This could explain the lack of clear responses of macroinvertebrate traits to improving ecological quality in certain sites or regions. Identifying shifts in the dispersal capacity of macroinvertebrate communities due to changes in ecological quality according to habitat type, different levels of anthropogenic pressure, or climatic conditions is a ripe topic for future investigation.

### 4.5 Concluding Remarks

Our results show that the dispersal capacity of the macroinvertebrate community, measured as *DCM*, as well as other dispersal‐related traits, can indicate changes in the ecological quality of freshwater ecosystems across broad spatial scales. However, heterogeneous responses across sites and countries suggest that such inferences must be made with caution, as the recovery of strong dispersers and sensitive taxa is likely to be influenced by a combination of other factors including regional extinctions, impoverished regional species pools, dispersal barriers, and interspecific competition. Maintaining and expanding monitoring initiatives, as well as incorporating the study of *DCM* and other dispersal‐related traits into management plans and biodiversity analyses, can help mitigate anthropogenic impacts on freshwater ecosystems through informing adaptive management strategies.

## Author Contributions

**Carlos Cano‐Barbacil:** conceptualization, data curation, formal analysis, investigation, methodology, writing – original draft. **James S. Sinclair:** data curation, formal analysis, methodology, writing – review and editing. **Ellen A. R. Welti:** data curation, writing – review and editing. **Peter Haase:** conceptualization, funding acquisition, writing – review and editing.

## Conflicts of Interest

The authors declare no conflicts of interest.

## Supporting information


Data S1.

## Data Availability

The data and model code support the findings of this study are openly available in Figshare at https://doi.org/10.6084/m9.figshare.25289308. Macroinvertebrate community data and site characteristics were obtained from the Knowledge Network for Biocomplexity repository at https://knb.ecoinformatics.org/view/doi:10.5063/F1NG4P4R and Figshare at https://doi.org/10.6084/m9.figshare.24486769.
