# Supplementary material for: Recovery and Degradation Drive Changes in the Dispersal Capacity of Stream Macroinvertebrate Communities
Source: Glob Chang Biol. 2025 Jan 27;31(1):e70054. doi: 10.1111/gcb.70054 (PMC11771701; doi:10.1111/gcb.70054)
Supplement: Supplementary file 1 — Data S1. [file GCB-31-e70054-s001.pdf]

Recovery and degradation drive changes in the dispersal capacity of stream macroinvertebrate communities

Carlos Cano-Barbacil[1,*], James S. Sinclair[1], Ellen A.R. Welti[2], Peter Haase[1,3]

[1] Senckenberg Research Institute and Natural History Museum Frankfurt, Department of River Ecology and Conservation, Gelnhausen, Germany

[2] Conservation Ecology Center, Smithsonian's National Zoo and Conservation Biology Institute, Front Royal, VA, USA

[3] Faculty of Biology, University of Duisburg-Essen, Essen, Germany

*Corresponding author. Address: Senckenberg Research Institute and Natural History Museum Frankfurt, Clamecystrasse 12, 63571 Gelnhausen, Germany. Email: Carlos.cano-barbacil@senckenberg.de

Table S1. Methods used to calculate the Ecological Quality Ratio (EQR) for each country. Modified from Sinclair et al. (2024).

Country	Method	Reference
Austria	Multimetric index using three metrics	(Ofenböck et al., 2004)
Belgium	Multimetric Macroinvertebrate Index Flanders	(Gabriels et al., 2010)
Bulgaria	Biotic Index	(Cheshmedjiev & Varadinova, 2013)
Cyprus	STAR Intercalibration Common Metric Index	(Feio et al., 2014)
Czechia	Multimetric index using river type-specific metrics	(Barešová et al., 2015; Mičaník et al., 2020)
Denmark	Danish Stream Fauna Index	(Andersen et al., 2016; Friberg et al., 2010)
Estonia	Multimetric index using five metrics	(Käiro et al., 2011)
Finland	Finnish Multimetric Index	(Andersen et al., 2016)
France	Global Biological Normalized Index	(Mondy et al., 2012)
Germany	Multimetric index using river type-specific metrics	(Berger et al., 2017)
Hungary	Hungarian Multimetric Index	(Boda et al., 2023; Bozóki et al., 2018)
Ireland	Quality Rating System	(Donohue et al., 2006)
Italy	STAR Intercalibration Common Metric Index	(Feio et al., 2014)
Latvia	Latvian Macroinvertebrate Index	(Ozoliņš et al., 2022; Ozoliņš & Skuja, 2016)
Lithuania	Lithuanian River Macroinvertebrate Index	(Lietuvos Respublikos aplinkos ministerija, 2007; Šidagytė-Copilas & Arbačiauskas, 2022)
Luxembourg	I_2M_2	(Mondy et al., 2012)
Netherlands	KRW-maatlatten	(Altenburg, 2018)
Norway	Poorest out of the Average Score Per Taxon index and the River Acidification Macroinvertebrate Index	(Andersen et al., 2016)
Portugal	South Portugal macroinvertebrate biotic index	(Leitão et al., 2014)
Spain	Iberian Biological Monitoring Working Party	(Ministerio de Agricultura, Alimentación y Medio Ambiente, 2015; Munné & Prat, 2009)
Sweden	Average Score Per Taxon and the DJ index	(Andersen et al., 2016; Bighiu et al., 2020; Dahl & Johnson, 2004)
Switzerland	Multimetric index following the German system for a type 3.2 river	(Berger et al., 2017)
United Kingdom	Whalley Hawkes Paisley Trigg (WHPT) Average Score Per Taxon and number of scored taxa	(Paisley et al., 2014; WFD-UKTAG, 2021)

Table S2. List of the nine dispersal-related traits divided into 40 categories retrieved from DISPERSE database (Sarremejane et al., 2020) and "freshwaterecology.info" (Schmidt-Kloiber & Hering, 2015). Modified from Sarremejane et al. (2020).

Trait	Category	Comments
Maximum body size (cm)	<0.25<br>≥0.25–0.5<br>≥0.5–1<br>≥1–2<br>≥2–4<br>≥4–8<br>≥8	Body size is well known to influence invertebrate's dispersal, especially for active dispersers. Larger species are able to disperse longer distances (Jenkins et al., 2007).
Female wing length (insects only) (mm)	<5<br>≥5–10<br>≥10–15<br>≥15–20<br>≥20–30<br>≥30–40<br>≥40–50<br>≥50	Female wing length is related to the adult insects' colonization capacity. Females with larger wings are likely to oviposit farther from their source population (Graham et al., 2017).
Wing pair type (insects only)	No wings<br>1 pair + halters<br>1 pair + small hind wings<br>1 pair + elytra or hemielytra<br>2 similar-sized pairs	Wing morphology, and in particular pair type, influence the dispersal capacity of flying adult insects (Sarremejane et al., 2020).
Life-cycle duration	≤1 year<br>>1 year	Longer life-cycle duration typically has more dispersal events (Stevens et al., 2013).
Adult life span	<1 week<br>≥1 week–1 month<br>≥1 month–1 year<br>≥1 year	Longer adult lifespan typically has more dispersal events (Stevens et al., 2013).
Lifelong fecundity (number of eggs per female)	<100<br>≥100–1000<br>≥1000–3000<br>≥3000	Greater fecundity typically increases the number of dispersal opportunities (Stevens et al., 2013).
Potential number of reproductive cycles per year	<1<br>1<br>>1	Greater number of annual reproductive events typically increase the propagule production (Stevens et al., 2013).
Dispersal strategy	Aquatic passive<br>Aquatic active<br>Aerial passive<br>Aerial active	Dispersal strategy represents the dispersal behaviour of species, and is used to calculate the dispersal capacity metric (*DCM*) (Li et al., 2018; Sarremejane et al., 2020).
Propensity to drift	Rare/catastrophic<br>Occasional<br>Frequent	Propensity to drift indicates the frequency of flow-mediated passive downstream dispersal events (Sarremejane et al., 2020).

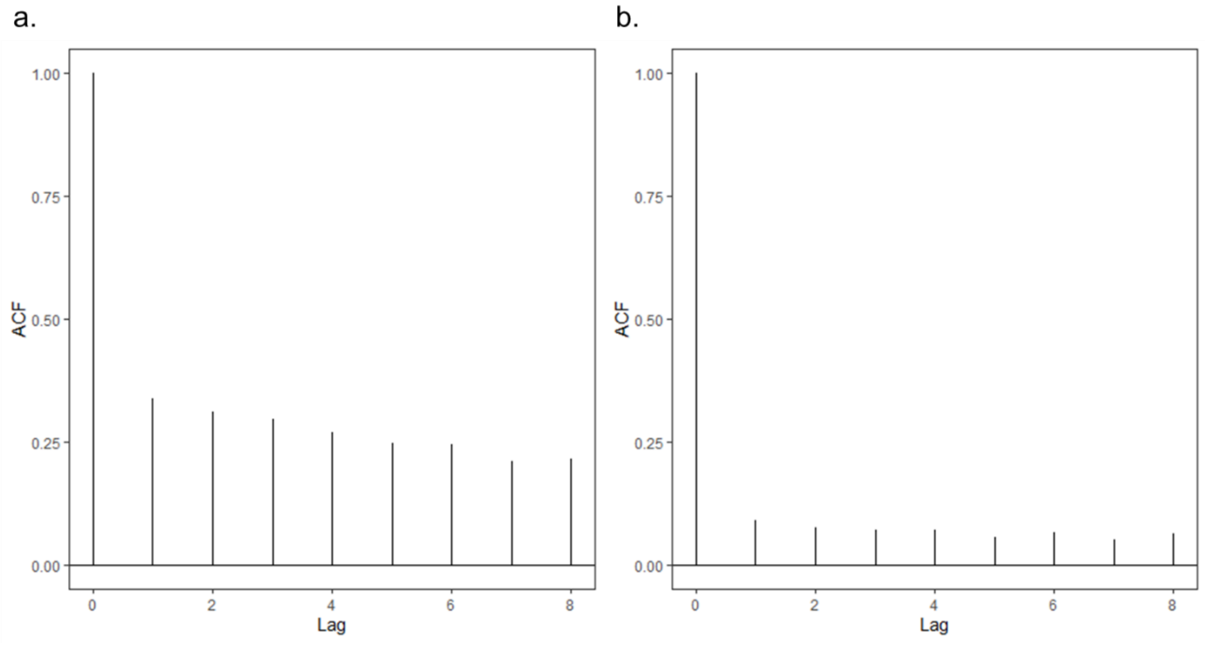

Figure S1. Autocorrelation plots. (a) Model without correlation structure showing certain temporal autocorrelation between consecutive observations; (b) model accounting for temporal autocorrelation by site showing very little or no correlation among samples.

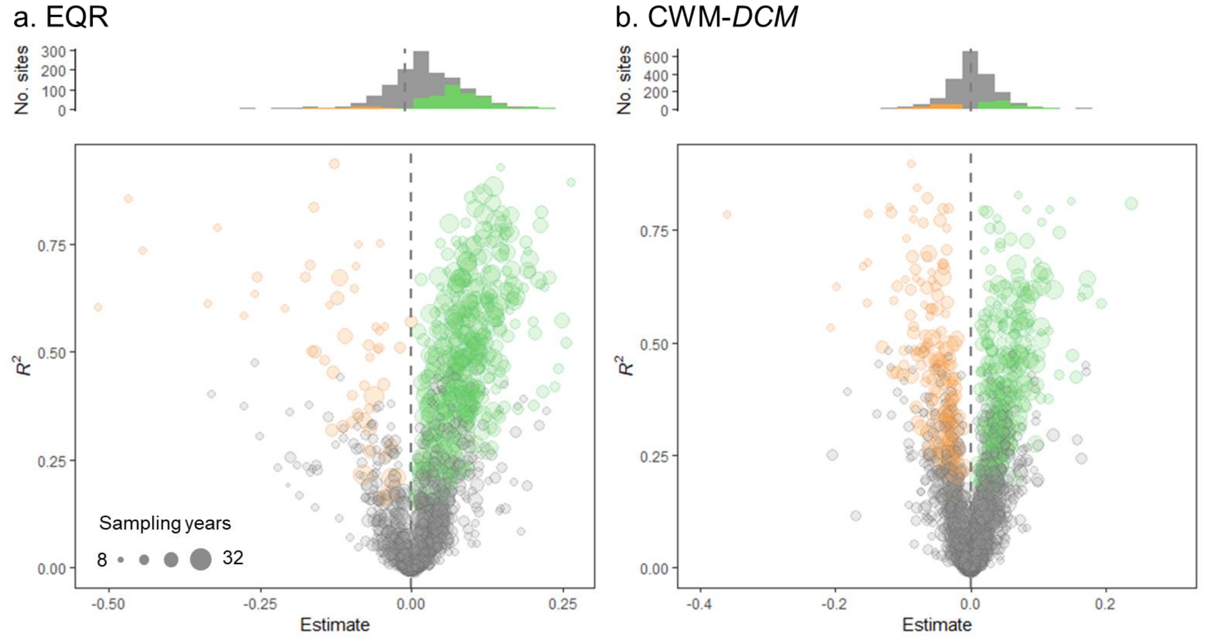

Figure S2. Coefficient of determination R^2 for (a) ecological quality ratio (EQR) and (b) dispersal capacity metric (CWM-*DCM*) plotted as a function of the slope estimates. The number of sampling years in a time series is visualised as bubble sizes. Bubbles are colour-coded as green (positive slope), orange (negative slope), and grey (non-significantly different from 0). Histograms show the distribution of slope estimates.

Table S3. Results of the linear mixed models. Marginal and conditional R^2, P-value of the random-effect term, and estimate, degrees of freedom (df), P-value and the corrected P-value (P_{fdr}) of the fixed-effect term are shown. *** indicates P <0.001.

Trait	Category	R^2_m	R^2_c	P_{rand}	Estimate	df	P	P_{fdr}
Dispersal capacity metric	-	8.7	26.7	***	0.172	15.2	**0.003**	**0.003**
Maximum body size (cm)	<0.25	1.8	16.0	***	-0.150	13.1	0.099	0.173
	≥0.25–0.5	0.1	6.3	0.062	-0.020	13.3	0.569	0.569
	≥0.5–1	2.2	13.6	0.125	0.105	11.9	**0.004**	**0.028**
	≥1–2	0.3	8.9	**0.003**	0.031	13.4	0.291	0.407
	≥2–4	0.1	2.5	0.252	0.032	11.6	0.536	0.569
	≥4–8	1.4	17.5	***	-0.160	15.0	**0.038**	0.089
	≥8	1.1	11.4	0.143	-0.111	10.9	**0.023**	0.081
Female wing length (insects only) (mm)	<5	6.5	22.3	***	-0.337	12.7	**0.002**	**0.004**
	≥5–10	6.4	15.3	***	0.279	13.7	**0.001**	**0.004**
	≥10–15	6.5	15.6	***	0.261	10.9	**0.002**	**0.004**
	≥15–20	2.3	9.9	0.946	0.146	10.4	***	***
	≥20–30	1.7	11.9	**0.004**	0.131	12.1	**0.015**	**0.024**
	≥30–40	0.1	7.9	**0.010**	0.023	11.2	0.548	0.731
	≥40–50	0.0	0.2	0.927	-0.001	35.4	0.877	0.877
	≥50	0.0	1.8	0.999	0.001	45.8	0.643	0.735
Wing pair type (insects only)	No wings	4.3	10.9	***	-0.302	13.5	**0.006**	**0.010**
	1 pair + halters	1.4	13.1	**0.002**	-0.155	13.7	**0.042**	**0.047**
	1 pair + small hind wings	10.1	15.6	***	0.389	11.6	***	***
	1 pair + elytra/hemielytra	1.2	3.5	***	0.124	14.2	**0.047**	**0.047**
	2 similar-sized pairs	10.0	16.0	0.093	0.358	8.1	***	***
Life-cycle duration	≤1 year	2.6	10.8	***	0.154	16.7	**0.014**	**0.014**
	>1 year	2.6	10.8	***	-0.154	16.7	**0.014**	**0.014**
Adult life span	<1 week	0.7	6.1	**0.003**	0.093	12.2	0.171	0.251
	≥1 week–1 month	7.1	16.0	**0.007**	0.294	11.5	***	**0.001**
	≥1 month–1 year	0.5	8.0	***	-0.064	11.5	0.251	0.251
	≥1 year	0.4	3.4	0.213	-0.081	6.5	0.219	0.251
Lifelong fecundity (number of eggs per female)	<100	1.6	11.9	***	-0.104	13.6	0.099	0.132
	≥100–1000	0.2	4.8	0.975	0.021	209.9	0.172	0.172
	≥1000–3000	3.8	6.8	***	0.156	10.5	**0.004**	**0.012**
	≥3000	4.1	8.4	***	0.210	12.3	**0.006**	**0.012**
Potential number of reproductive cycles per year	<1	3.3	16.7	0.130	0.209	9.3	**0.002**	**0.002**
	1	6.3	18.0	**0.010**	0.184	9.3	**0.002**	**0.002**
	>1	7.7	22.7	***	-0.215	10.9	***	**0.002**
Dispersal strategy	Aquatic passive	7.6	25.1	***	-0.124	15.6	***	***
	Aquatic active	0.4	24.9	***	0.018	13.3	0.327	0.327
	Aerial passive	1.9	9.1	***	0.098	15.7	**0.034**	**0.045**
	Aerial active	10.6	17.1	***	0.239	13.9	***	***
Propensity to drift	Rare/catastrophic	0.9	8.5	***	-0.081	12.9	0.142	0.213
	Occasional	0.2	8.7	***	0.029	16.4	0.470	0.470
	Frequent	1.8	7.6	**0.010**	0.109	10.1	**0.033**	0.099

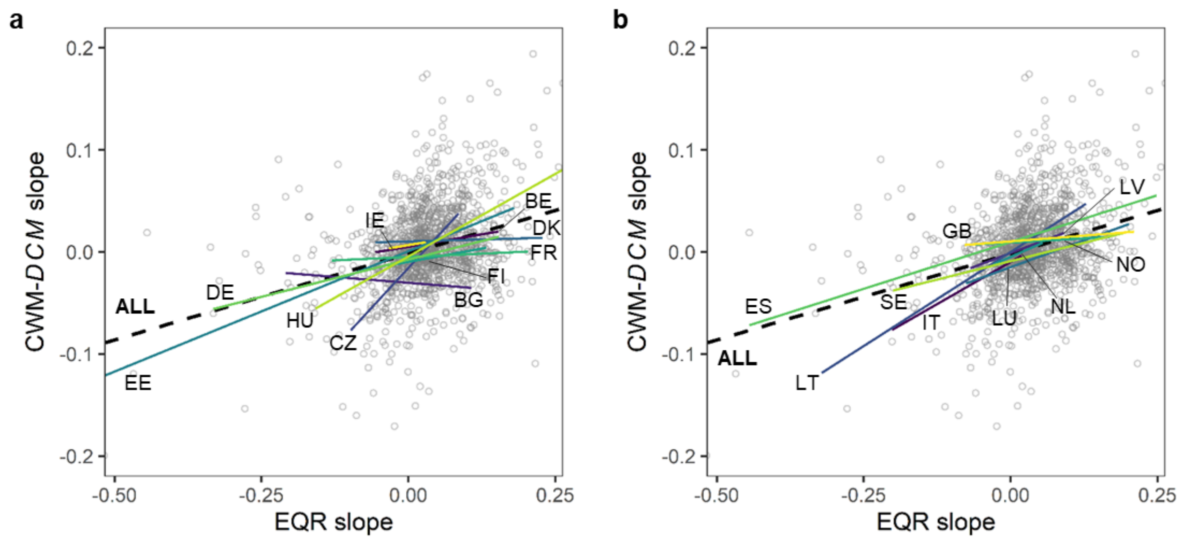

Figure S3. Relationship between changes in the dispersal capacity metric of the community (CWM-*DCM* slope) and changes in the ecological quality ratio (EQR slope) across countries. Data points are represented by grey circles and models are fitted by solid lines. The black dashed line corresponds to a linear model in which a single slope was fitted to all countries (ALL). Note that trends for countries with less than three time series have not been represented, although they were included in the calculation of the overall trend. (a) BE = Belgium; BG = Bulgaria; CZ = Czechia; DK = Denmark; EE = Estonia; FI = Finland; FR = France; DE = Germany; HU = Hungary; IE = Ireland. (b) IT = Italy; LV = Latvia; LT = Lithuania; LU = Luxembourg; NL = Netherlands; NO = Norway; ES = Spain; SE = Sweden; GB = United Kingdom.

Table S4. Results of linear mixed models evaluating trait group responses to EQR. Marginal and conditional R^2, *P*-value of the random-effect term (P_{rand}), and *P*-value of the fixed-effect terms are shown. Results can be interpreted with Table S5. sp = species groups (i.e. new, remaining or extirpated species); EQR = site groups (i.e. recovering, stable or degrading sites); int = interaction between both factors. *** indicates *P* <0.001.

Trait	Category	R^2_m	R^2_c	P_{rand}	P_{sp}	P_{EQR}	P_{int}
Dispersal capacity metric	-	3.5	27.2	***	**0.018**	***	***
Maximum body size (cm)	<0.25	1.4	1.4	1.000	**0.015**	0.080	0.237
	≥0.25–0.5	2.5	8.4	***	***	0.629	***
	≥0.5–1	0.6	3.7	**0.049**	0.315	0.275	**0.018**
	≥1–2	1.3	1.3	1.000	**0.049**	0.429	0.336
	≥2–4	0.3	8.7	***	0.273	0.281	0.165
	≥4–8	0.8	0.8	1.000	**0.016**	0.068	0.124
	≥8	0.2	17.5	***	0.961	0.150	0.512
Female wing length (insects only) (mm)	<5	2.6	26.0	***	***	***	**0.017**
	≥5–10	2.7	17.6	***	***	**0.001**	***
	≥10–15	1.5	24.9	***	**0.036**	**0.007**	***
	≥15–20	0.7	0.7	1.000	**0.036**	0.436	***
	≥20–30	0.8	12.5	***	0.959	0.191	***
	≥30–40	2.2	14.3	***	**0.019**	***	***
	≥40–50	2.0	7.0	**0.002**	***	0.100	**0.020**
	≥50	0.9	2.5	0.315	0.068	0.387	0.328
Wing pair type (insects only)	No wings	5.3	41.1	***	***	***	***
	1 pair + halters	2.5	27.5	***	***	***	**0.002**
	1 pair + small hind wings	6.4	14.6	***	***	***	***
	1 pair + elytra or hemielytra	4.8	25.9	***	***	0.234	***
	2 similar-sized pairs	7.9	42.1	***	***	***	***
Life-cycle duration	≤1 year	1.0	13.8	***	**0.005**	0.571	**0.034**
	>1 year	1.0	13.8	***	**0.005**	0.571	**0.034**
Adult life span	<1 week	2.8	24.3	***	***	0.248	***
	≥1 week–1 month	4.4	24.1	***	0.168	***	***
	≥1 month–1 year	0.8	13.9	***	**0.017**	**0.027**	0.091
	≥1 year	1.8	20.9	***	***	0.158	***
Lifelong fecundity (number of eggs per female)	<100	1.1	18.6	***	0.069	***	**0.012**
	≥100–1000	1.1	6.0	**0.002**	**0.027**	0.427	***
	≥1000–3000	2.2	12.9	***	**0.024**	***	***
	≥3000	1.6	19.2	***	**0.032**	***	**0.049**
Potential number of reproductive cycles per year	<1	2.9	24.6	***	0.505	***	***
	1	4.2	20.7	***	***	0.989	0.429
	>1	4.8	35.7	***	***	0.106	0.450
Dispersal strategy	Aquatic passive	13.7	31.5	***	***	0.314	***
	Aquatic active	2.3	15.6	***	***	0.181	0.730
	Aerial passive	3.9	20.8	***	***	**0.030**	**0.032**
	Aerial active	11.1	34.6	***	***	***	***
Propensity to drift	Rare/catastrophic	7.0	45.6	***	***	0.372	***
	Occasional	5.7	43.7	***	***	0.270	***
	Frequent	9.9	41.8	***	***	0.659	***

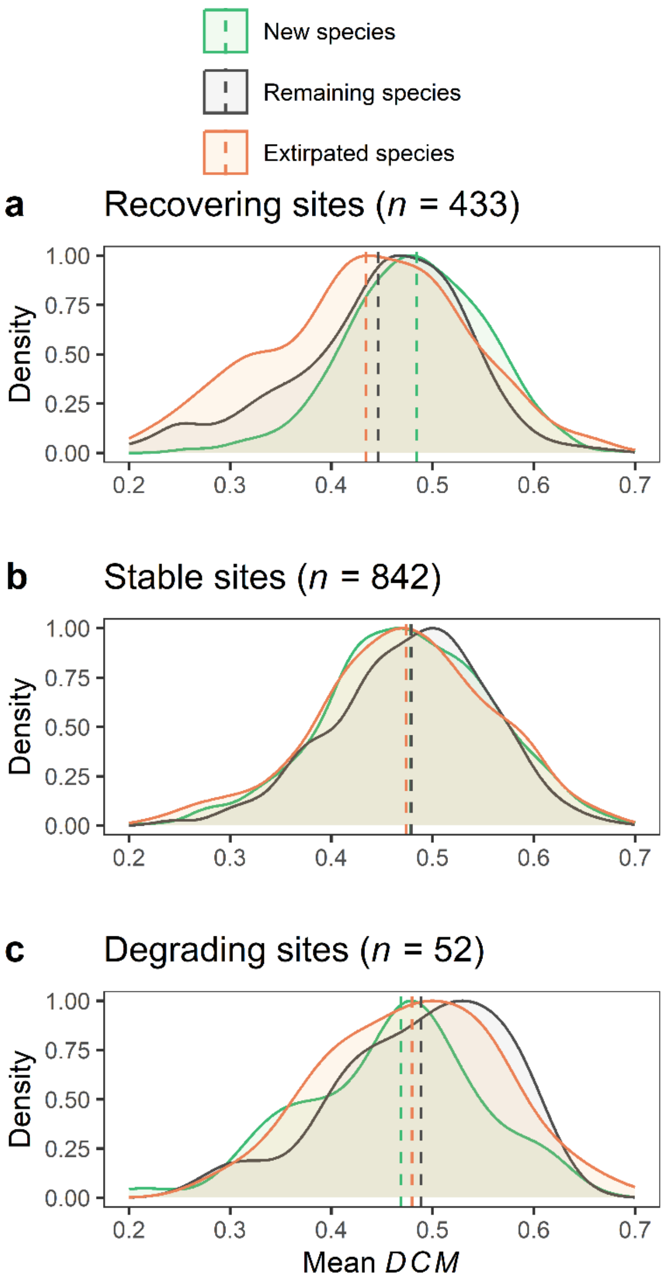

Figure S4. Scaled kernel density estimation for mean dispersal capacity metric (*DCM*) of new, remaining and extirpated species in (a) recovering sites (increasing ecological quality); (b) stable sites (no significant changes in ecological quality); and (c) degrading sites (decreasing ecological quality).

Table S5. Mean trait values of new (N)—not present initially and then present later—, remaining (R)—no change—and extirpated (E)—present and then not present—species in recovering, stable and degrading sites. Colder colours indicate higher values while warmer colours indicate lower values; white cells indicate intermediate values within each trait category.

Trait	Category	Recovering sites			Stable sites			Degrading sites		
		N	R	E	N	R	E	N	R	E
Dispersal capacity metric	-	0.484	0.446	0.434	0.478	0.479	0.474	0.469	0.489	0.480
Maximum body size (cm)	<0.25	0.012	0.008	0.008	0.013	0.009	0.009	0.012	0.011	0.011
	≥0.25–0.5	0.127	0.127	0.110	0.122	0.122	0.116	0.114	0.137	0.112
	≥0.5–1	0.377	0.351	0.358	0.367	0.365	0.362	0.376	0.372	0.374
	≥1–2	0.275	0.300	0.283	0.280	0.300	0.287	0.290	0.279	0.281
	≥2–4	0.164	0.170	0.173	0.164	0.162	0.168	0.151	0.155	0.168
	≥4–8	0.035	0.036	0.055	0.040	0.033	0.043	0.041	0.036	0.041
	≥8	0.010	0.009	0.012	0.015	0.010	0.015	0.016	0.011	0.012
Female wing length (insects only) (mm)	<5	0.365	0.443	0.444	0.358	0.401	0.360	0.351	0.416	0.356
	≥5–10	0.328	0.264	0.272	0.326	0.285	0.306	0.354	0.284	0.310
	≥10–15	0.151	0.164	0.140	0.159	0.170	0.167	0.146	0.157	0.159
	≥15–20	0.064	0.065	0.072	0.066	0.065	0.078	0.063	0.071	0.075
	≥20–30	0.051	0.040	0.051	0.051	0.047	0.055	0.045	0.048	0.060
	≥30–40	0.027	0.019	0.009	0.028	0.028	0.025	0.027	0.018	0.026
	≥40–50	0.012	0.004	0.009	0.009	0.003	0.008	0.012	0.005	0.011
	≥50	0.003	0.001	0.003	0.002	0.001	0.002	0.001	0.001	0.003
Wing pair type (insects only)	No wings	0.193	0.357	0.297	0.224	0.278	0.224	0.267	0.243	0.209
	1 pair + halters	0.242	0.251	0.292	0.205	0.231	0.214	0.210	0.255	0.207
	1 pair + small hind wings	0.083	0.099	0.053	0.093	0.105	0.084	0.108	0.125	0.092
	1 pair + elytra/hemielytra	0.143	0.077	0.170	0.153	0.097	0.168	0.127	0.101	0.144
	2 similar-sized pairs	0.340	0.217	0.189	0.325	0.289	0.309	0.288	0.276	0.348
Life-cycle duration	≤1 year	0.689	0.645	0.644	0.672	0.660	0.658	0.676	0.678	0.665
	>1 year	0.311	0.355	0.356	0.328	0.340	0.342	0.324	0.322	0.335
Adult life span	<1 week	0.312	0.304	0.268	0.291	0.308	0.270	0.316	0.360	0.286
	≥1 week–1 month	0.228	0.169	0.172	0.218	0.209	0.216	0.188	0.203	0.237
	≥1 month–1 year	0.334	0.342	0.365	0.341	0.315	0.353	0.344	0.278	0.323
	≥1 year	0.125	0.186	0.195	0.150	0.168	0.160	0.152	0.159	0.154
Lifelong fecundity (number of eggs per female)	<100	0.321	0.364	0.375	0.331	0.333	0.334	0.324	0.342	0.323
	≥100–1000	0.544	0.494	0.496	0.515	0.505	0.509	0.499	0.485	0.514
	≥1000–3000	0.106	0.116	0.105	0.116	0.127	0.123	0.144	0.137	0.130
	≥3000	0.029	0.027	0.024	0.037	0.034	0.035	0.033	0.036	0.033
Potential no. of reproductive cycles per year	<1	0.088	0.067	0.066	0.085	0.078	0.086	0.079	0.082	0.094
	1	0.633	0.592	0.632	0.635	0.589	0.632	0.615	0.588	0.643
	>1	0.279	0.342	0.302	0.280	0.332	0.282	0.305	0.330	0.262
Dispersal strategy	Aquatic passive	0.310	0.408	0.336	0.327	0.384	0.321	0.351	0.369	0.325
	Aquatic active	0.290	0.274	0.292	0.286	0.269	0.291	0.285	0.262	0.291
	Aerial passive	0.107	0.113	0.100	0.100	0.109	0.094	0.102	0.116	0.095
	Aerial active	0.292	0.205	0.272	0.287	0.238	0.294	0.262	0.253	0.289
Propensity to drift	Rare/catastrophic	0.693	0.645	0.750	0.710	0.639	0.722	0.724	0.631	0.704
	Occasional	0.219	0.235	0.178	0.206	0.243	0.198	0.191	0.255	0.213
	Frequent	0.088	0.120	0.071	0.084	0.118	0.080	0.085	0.114	0.083

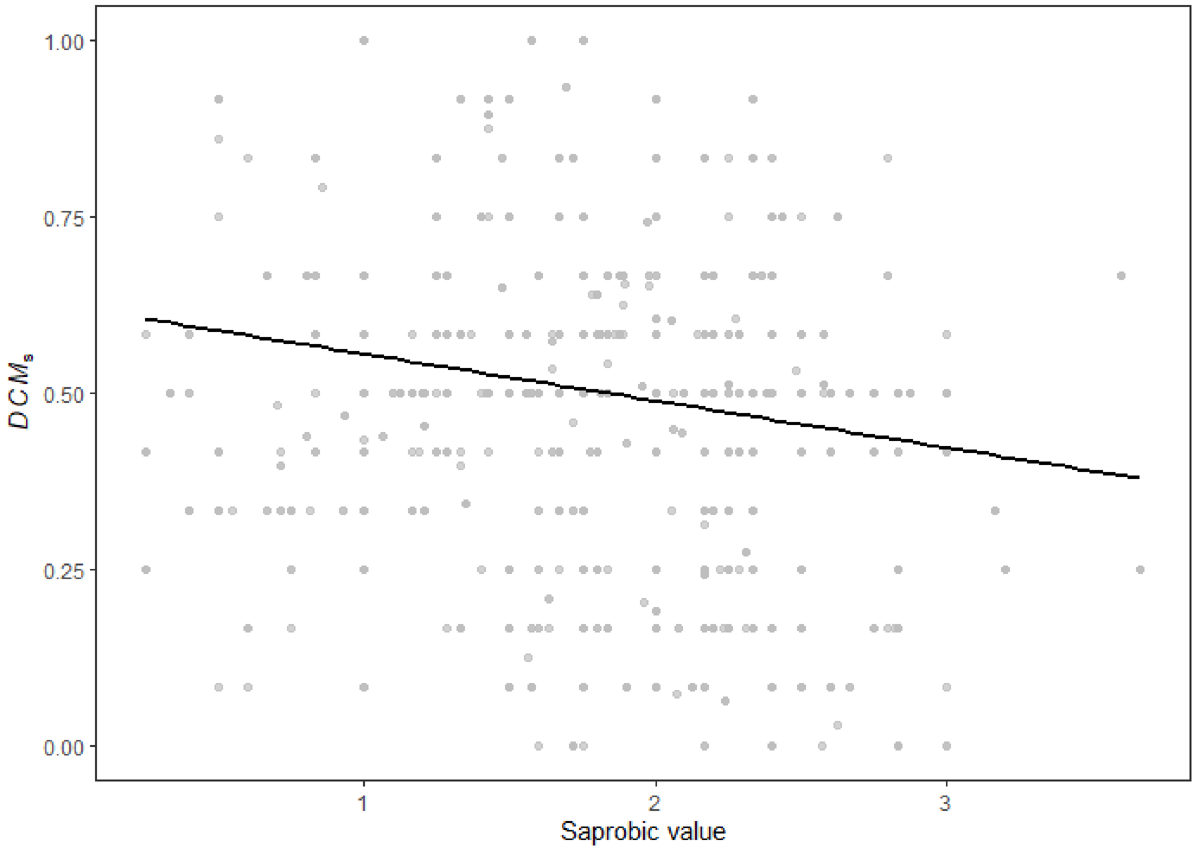

Figure S5. Correlation between the dispersal capacity metric (*DCM*$_s$) and the saprobic value of each macroinvertebrate taxon (r = -0.166, P < 0.001). The saprobic value is an estimate of tolerance level to organic pollution, ranging from 0 (species occurring in perfectly clean water courses) to 4 (species occurring in heavily polluted water courses). It was calculated following the methodology suggested by Zahradkova and Soldan (2008).

Appendix S1

The results from the analysis, which included only sites where the first and last three years were consecutive, were consistent with those from the full dataset, despite a 61.6% reduction in the number of time series. In the original analysis we evaluated 433 recovering, 842 stable and 52 degrading sites, while now we have analysed 152, 331 and 26 sites respectively. Similar to previous results, we found that recovering sites experienced a net gain in taxa due to the arrival and establishment of new taxa, while degrading sites showed a net loss of taxa (see comparison in Table S6). More specifically, 38.0% of the taxa recorded in the last three sampling years at recovering sites were new compared to the first three sampling years, while 27.3% remained the same and 34.7% disappeared, on average. In stable sites, 31.3% of the taxa were new, 40.1% remained the same, and 28.6% disappeared. By contrast, in degrading sites, only 27.1% of the taxa were new, while 39.4% remained unchanged, and 33.5% disappeared.

In accordance with the results obtained from the full dataset, we found that taxa with stronger dispersal capacity tended to replace taxa with weaker dispersal capacity in recovering sites (Figure S6). In addition, new taxa in recovering sites had similar mean *DCM* to new taxa in degrading sites. However, extirpated taxa in recovering sites had a lower mean *DCM* compared to higher *DCM*s of extirpated taxa in degrading sites (interaction $P < 0.001$). In sites with no clear EQR trends, new, remaining, and extirpated taxa showed relatively similar mean *DCM*. In agreement with previous results, we found that remaining species in degrading sites have the highest dispersal capacity, suggesting that high dispersal capacity may be beneficial for stable occurrence under occasional unfavourable ecological quality conditions. The results of the linear mixed model revealed heterogeneous responses across sites ($P_{rand} < 0.001$), with fixed effects explaining 3.4% of the variation, while the variation explained with the model also including random effects (i.e. site) increased to 27.0%.

Similar to the analysis of the full dataset, the results indicated that new taxa in recovering sites showed traits reflecting higher dispersal capacity (e.g. aerial and aquatic active dispersers, insects with two similar-sized pairs of wings or with larger wings, frequent propensity to drift) than extirpated taxa (Table S7). In contrast, extirpated taxa in degrading sites tend to show traits reflecting higher dispersal capacity (e.g. aquatic active dispersers, insects with two similar-sized pairs of wings) than new taxa.

Table S6. Comparison of the proportion (%) of new, remaining, and extirpated taxa across recovering, stable, and degrading sites for both datasets (i.e. full dataset including all time series and the subset containing time series with consecutive first and last three years).

	All time series			***Time series with consecutive first and last three years***		
	**New**	**Remaining**	**Extirpated**	**New**	**Remaining**	**Extirpated**
**Recovering**	38.0	27.3	34.7	41.6	31.4	27.0
**Stable**	31.3	40.1	28.6	28.8	42.5	28.7
**Degrading**	27.1	39.4	33.5	22.3	41.4	36.4

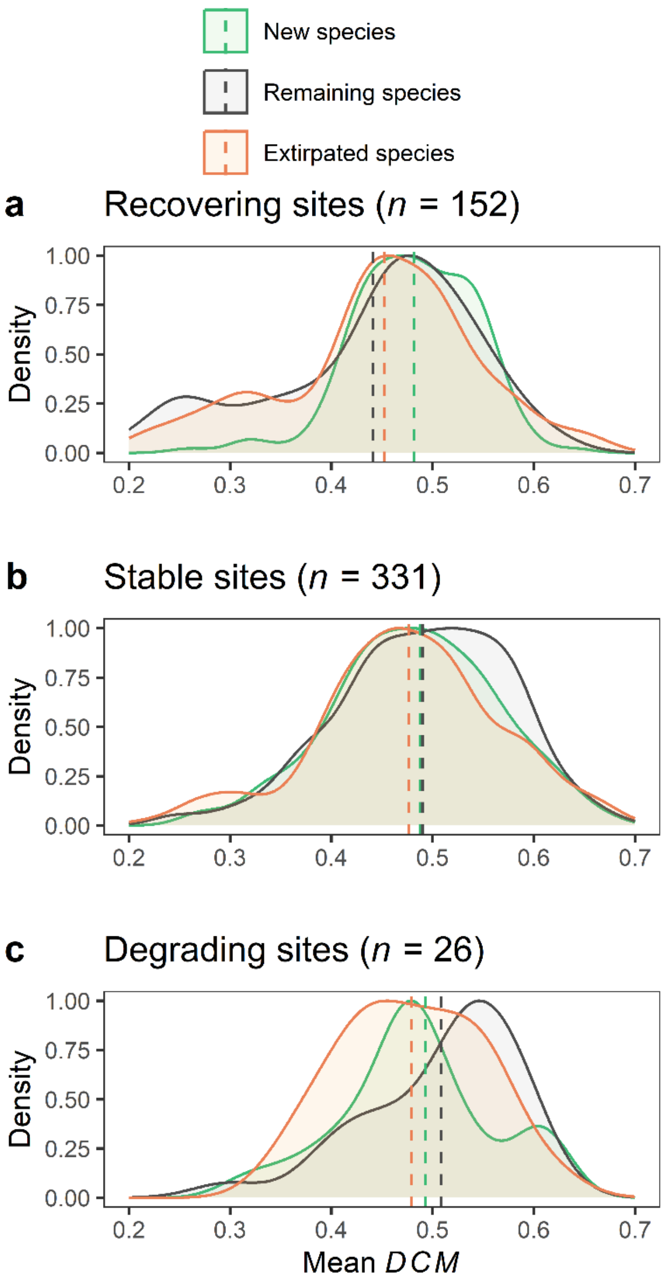

Figure S6. Scaled kernel density estimation for mean dispersal capacity metric (*DCM*) of new, remaining and extirpated species in (a) recovering sites (increasing ecological quality); (b) stable sites (no significant changes in ecological quality); and (c) degrading sites (decreasing ecological quality)

Table S7. Mean trait values of new (N)—not present initially and then present later—, remaining (R)—no change—and extirpated (E)—present and then not present—species in recovering, stable and degrading sites. Colder colours indicate higher values while warmer colours indicate lower values; white cells indicate intermediate values within each trait category. Only traits with $R^2_m > 5\%$ are shown.

Trait	Category	Recovering sites			Stable sites			Degrading sites		
		N	R	E	N	R	E	N	R	E
Wing pair type (insects only)	1 pair + halters	0.215	0.258	0.271	0.157	0.243	0.193	0.170	0.253	0.172
	1 pair + elytra/hemielytra	0.152	0.066	0.155	0.162	0.101	0.160	0.147	0.116	0.168
	2 similar-sized pairs	0.346	0.212	0.211	0.351	0.303	0.322	0.343	0.312	0.399
Potential no. of reproductive cycles per year	<1	0.100	0.065	0.073	0.098	0.076	0.087	0.103	0.085	0.104
	>1	0.263	0.336	0.285	0.272	0.325	0.280	0.281	0.305	0.251
Dispersal strategy	Aquatic passive	0.310	0.429	0.335	0.326	0.381	0.328	0.326	0.362	0.319
	Aquatic active	0.291	0.261	0.290	0.287	0.261	0.290	0.293	0.257	0.295
	Aerial active	0.298	0.193	0.271	0.290	0.247	0.288	0.282	0.268	0.269
Propensity to drift	Rare/catastrophic	0.703	0.655	0.749	0.705	0.634	0.714	0.702	0.606	0.707
	Frequent	0.084	0.116	0.071	0.086	0.121	0.080	0.092	0.122	0.076

References

Altenburg, W. (2018). *Referenties en maatlatten voor natuurlijke watertypen voor de Kaderrichtlijn Water 2021-2027*. https://library.wur.nl/WebQuery/wurpubs/547103

Andersen, J. H., Aroviita, J., Carstensen, J., Friberg, N., Johnson, R. K., Kauppila, P., Lindegarth, M., Murray, C., & Norling, K. (2016). Approaches for integrated assessment of ecological and eutrophication status of surface waters in Nordic Countries. *Ambio*, *45*(6), 681–691. https://doi.org/10.1007/s13280-016-0767-8

Barešová, L., Němejcová, D., Zahradkova, S., & Kokeš, J. (2015). *Assessment method for ecological status of rivers based on biological quality element benthic invertebrates in the Czech Republic*.

Berger, E., Haase, P., Kuemmerlen, M., Leps, M., Schäfer, R. B., & Sundermann, A. (2017). Water quality variables and pollution sources shaping stream macroinvertebrate communities. *Science of The Total Environment*, *587–588*, 1–10. https://doi.org/10.1016/j.scitotenv.2017.02.031

Bighiu, M. A., Höss, S., Traunspurger, W., Kahlert, M., & Goedkoop, W. (2020). Limited effects of pesticides on stream macroinvertebrates, biofilm nematodes, and algae in intensive agricultural landscapes in Sweden. *Water Research*, *174*, 115640. https://doi.org/10.1016/j.watres.2020.115640

Boda, P., Várbíró, G., & Ficsór, M. (2023). *Módszertani Kézikönyv a Víz Keretirányelv feladataihoz kapcsolódóan a makroszkopikus vízi gerinctelenek mintavételéhez és ökológiai állapotértékeléséhez*. Ökológiai Kutatóközpont, Budapest, 60 oldal.

Bozóki, T., Krasznai-Kun, E. Á., Csercsa, A., Várbíró, G., & Boda, P. (2018). Temporal and spatial dynamics in aquatic macroinvertebrate communities along a small urban stream. *Environmental Earth Sciences*, *77*(15), 559. https://doi.org/10.1007/s12665-018-7735-5

Cheshmedjiev, S., & Varadinova, E. (2013). Bottom macroinvertabrates. In D. Belkinova (Ed.), *Biological analysis and ecological assessment of surface water types in Bulgaria* (pp. 147–162). P. Hilendarski University Publishing House.

Dahl, J., & Johnson, R. K. (2004). A multimetric macroinvertebrate index for detecting organic pollution of streams in southern Sweden. *Archiv Für Hydrobiologie*, *160*(4), 487–513. https://doi.org/10.1127/0003-9136/2004/0160-0487

Donohue, I., McGarrigle, M. L., & Mills, P. (2006). Linking catchment characteristics and water chemistry with the ecological status of Irish rivers. *Water Research*, *40*(1), 91–98. https://doi.org/10.1016/j.watres.2005.10.027

Feio, M. J., Ferreira, J., Buffagni, A., Erba, S., Dörflinger, G., Ferréol, M., Munné, A., Prat, N., Tziortzis, I., & Urbanič, G. (2014). Comparability of ecological quality boundaries in the Mediterranean basin using freshwater benthic invertebrates. Statistical options and implications. *Science of The Total Environment*, *476–477*, 777–784. https://doi.org/10.1016/j.scitotenv.2013.07.085

Friberg, N., Skriver, J., Larsen, S. E., Pedersen, M. L., & Buffagni, A. (2010). Stream macroinvertebrate occurrence along gradients in organic pollution and eutrophication. *Freshwater Biology*, *55*(7), 1405–1419. https://doi.org/10.1111/j.1365-2427.2008.02164.x

Gabriels, W., Lock, K., De Pauw, N., & Goethals, P. L. M. (2010). Multimetric Macroinvertebrate Index Flanders (MMIF) for biological assessment of rivers and lakes in Flanders (Belgium). *Limnologica - Ecology and Management of Inland Waters*, *40*(3), 199–207. https://doi.org/10.1016/j.limno.2009.10.001

Graham, S. E., Storey, R., & Smith, B. (2017). Dispersal distances of aquatic insects: Upstream crawling by benthic EPT larvae and flight of adult Trichoptera along valley floors. *New Zealand Journal of Marine and Freshwater Research*, *51*(1), 146–164. https://doi.org/10.1080/00288330.2016.1268175

Jenkins, D. G., Brescacin, C. R., Duxbury, C. V., Elliott, J. A., Evans, J. A., Grablow, K. R., Hillegass, M., Lyon, B. N., Metzger, G. A., Olandese, M. L., Pepe, D., Silvers, G. A., Suresch, H. N., Thompson, T. N., Trexler, C. M., Williams, G. E., Williams, N. C., & Williams, S. E. (2007). Does size matter for dispersal distance? *Global Ecology and Biogeography*, *16*(4), 415–425. https://doi.org/10.1111/j.1466-8238.2007.00312.x

Käiro, K., Möls, T., Timm, H., Virro, T., & Järvekülg, R. (2011). The effect of damming on biological quality according to macroinvertebrates in some Estonian streams, Central—Baltic Europe: A pilot study. *River Research and Applications*, *27*(7), 895–907. https://doi.org/10.1002/rra.1406

Leitão, F., Hughes, S. J., Máximo, I., Atanasova, N., Furtado, A., & Chicharo, L. (2014). Habitat-Oriented Sampling of Macroinvertebrates Affects the Determination of Ecological Status in Temporary Mediterranean River Systems. *River Research and Applications*, *30*(10), 1233–1247. https://doi.org/10.1002/rra.2800

Li, F., Tonkin, J. D., & Haase, P. (2018). Dispersal capacity and broad-scale landscape structure shape benthic invertebrate communities along stream networks. *Limnologica*, *71*, 68–74. https://doi.org/10.1016/j.limno.2018.06.003

Lietuvos Respublikos aplinkos ministerija. (2007). *Dėl Paviršinių vandens telkinių būklės nustatymo metodikos patvirtinimo*. https://e-seimas.lrs.lt/portal/legalAct/lt/TAD/TAIS.296626/asr

Mičaník, T., Vyskoč, P., Prchalová, H., Polášek, M., Němejcová, D., Durčák, M., & Richter, P. (2020). Hodnocení stavu útvarů povrchových vod v České republice pro 3. Plánovací období plánů povodí. *Vodohospodářské technicko-ekonomické informace*, *62*(6), 4–18.

Ministerio de Agricultura, Alimentación y Medio Ambiente. (2015). Real Decreto 817/2015, de 11 de septiembre, por el que se establecen los criterios de seguimiento y evaluación del estado de las aguas superficiales y las normas de calidad ambiental. In *Boletín Oficial del Estado número 219* (pp. 80582–80677). https://www.boe.es/eli/es/rd/2015/09/11/817

Mondy, C. P., Villeneuve, B., Archaimbault, V., & Usseglio-Polatera, P. (2012). A new macroinvertebrate-based multimetric index (I2M2) to evaluate ecological quality of French wadeable streams fulfilling the WFD demands: A taxonomical and trait approach. *Ecological Indicators*, *18*, 452–467. https://doi.org/10.1016/j.ecolind.2011.12.013

Munné, A., & Prat, N. (2009). Use of macroinvertebrate-based multimetric indices for water quality evaluation in Spanish Mediterranean rivers: An intercalibration approach with the IBMWP index. *Hydrobiologia*, *628*(1), 203–225. https://doi.org/10.1007/s10750-009-9757-1

Ofenböck, T., Moog, O., Gerritsen, J., & Barbour, M. (2004). A Stressor Specific Multimetric Approach for Monitoring Running Waters in Austria Using Benthic Macro-Invertebrates. In D. Hering, P. F. M. Verdonschot, O. Moog, & L. Sandin (Eds.), *Integrated Assessment of Running Waters in Europe* (pp. 251–268). Springer Netherlands. https://doi.org/10.1007/978-94-007-0993-5_15

Ozoliņš, D., Karklina, I., Skuja, A., Uzule, L., Kokorite, I., Medne-Peipere, M., & Lazdiņš, A. (2022). The Impact of Forest Fertilization on the Ecological Quality of Two Hemiboreal Streams. *Forests*, *13*(2), Article 2. https://doi.org/10.3390/f13020196

Ozoliņš, D., & Skuja, A. (2016). *Fitting the new Latvian Macroinvertebrate Index (LMI) for rivers to the results of the Central-Baltic Geographical Intercalibration Group*.

Paisley, M. F., Trigg, D. J., & Walley, W. J. (2014). Revision of the Biological Monitoring Working Party (bmwp) Score System: Derivation of Present-Only and Abundance-Related Scores from Field Data. *River Research and Applications*, *30*(7), 887–904. https://doi.org/10.1002/rra.2686

Sarremejane, R., Cid, N., Stubbington, R., Datry, T., Alp, M., Cañedo-Argüelles, M., Cordero-Rivera, A., Csabai, Z., Gutiérrez-Cánovas, C., Heino, J., Forcellini, M., Millán, A., Paillex, A., Pařil, P., Polášek, M., Tierno de Figueroa, J. M., Usseglio-Polatera, P., Zamora-Muñoz, C., & Bonada, N. (2020). DISPERSE, a trait database to assess the dispersal potential of European aquatic macroinvertebrates. *Scientific Data*, *7*(1), Article 1. https://doi.org/10.1038/s41597-020-00732-7

Schmidt-Kloiber, A., & Hering, D. (2015). www.freshwaterecology.info – An online tool that unifies, standardises and codifies more than 20,000 European freshwater organisms and their ecological preferences. *Ecological Indicators*, *53*, 271–282. https://doi.org/10.1016/j.ecolind.2015.02.007

Šidagytė-Copilas, E., & Arbačiauskas, K. (2022). A multimetric macroinvertebrate index for the assessment of the ecological status of Lithuanian rivers. *Limnologica*, *97*, 126010. https://doi.org/10.1016/j.limno.2022.126010

Sinclair, J. S., Welti, E. A. R., Altermatt, F., Álvarez-Cabria, M., Aroviita, J., Baker, N. J., Barešová, L., Barquín, J., Bonacina, L., Bonada, N., Cañedo-Argüelles, M., Csabai, Z., de Eyto, E., Dohet, A.,

Dörflinger, G., Eriksen, T. E., Evtimova, V., Feio, M. J., Ferréol, M., … Haase, P. (2024). Multi-decadal improvements in the ecological quality of European rivers are not consistently reflected in biodiversity metrics. *Nature Ecology & Evolution*, *8*(3), 430–441. https://doi.org/10.1038/s41559-023-02305-4

Stevens, V. M., Trochet, A., Blanchet, S., Moulherat, S., Clobert, J., & Baguette, M. (2013). Dispersal syndromes and the use of life-histories to predict dispersal. *Evolutionary Applications*, *6*(4), 630–642. https://doi.org/10.1111/eva.12049

WFD-UKTAG. (2021). *UKTAG River Assessment Method Benthic Invertebrate Fauna. Invertebrates (General Degradation): Walley, Hawkes, Paisley & Trigg (WHPT) metric in River Invertebrate Classification Tool (RICT)*. Water Framework Directive – United Kingdom Advisory Group.

Zahradkova, S., & Soldan, T. (2008). Saprobic System. In S. E. Jørgensen & B. D. Fath (Eds.), *Encyclopedia of Ecology* (pp. 3141–3143). Academic Press. https://doi.org/10.1016/B978-008045405-4.00130-0
